# A deep learning model for molecular label transfer that enables cancer cell identification from histopathology images

**DOI:** 10.1038/s41698-022-00252-0

**Published:** 2022-03-02

**Authors:** Andrew Su, HoJoon Lee, Xiao Tan, Carlos J. Suarez, Noemi Andor, Quan Nguyen, Hanlee P. Ji

**Affiliations:** 1 Institute for Molecular Bioscience, The University of Queensland, Brisbane, QLD 4072 Australia; 2 Division of Oncology, Department of Medicine, Stanford University School of Medicine, Stanford, CA 94305 USA; 3 Department of Pathology, Stanford University School of Medicine, Stanford, CA 94305 USA; 4 Stanford Genome Technology Center, Stanford University, Palo Alto, CA 94304 USA; 5 Present Address: Department of Integrated Mathematical Oncology, Moffitt Cancer Center, 12902 Magnolia Drive, Tampa, FL 33612 USA

**Keywords:** Colon cancer, Tumour heterogeneity

## Abstract

Deep-learning classification systems have the potential to improve cancer diagnosis. However, development of these computational approaches so far depends on prior pathological annotations and large training datasets. The manual annotation is low-resolution, time-consuming, highly variable and subject to observer variance. To address this issue, we developed a method, H&E Molecular neural network (HEMnet). HEMnet utilizes immunohistochemistry as an initial molecular label for cancer cells on a H&E image and trains a cancer classifier on the overlapping clinical histopathological images. Using this molecular transfer method, HEMnet successfully generated and labeled 21,939 tumor and 8782 normal tiles from ten whole-slide images for model training. After building the model, HEMnet accurately identified colorectal cancer regions, which achieved 0.84 and 0.73 of ROC AUC values compared to p53 staining and pathological annotations, respectively. Our validation study using histopathology images from TCGA samples accurately estimated tumor purity, which showed a significant correlation (regression coefficient of 0.8) with the estimation based on genomic sequencing data. Thus, HEMnet contributes to addressing two main challenges in cancer deep-learning analysis, namely the need to have a large number of images for training and the dependence on manual labeling by a pathologist. HEMnet also predicts cancer cells at a much higher resolution compared to manual histopathologic evaluation. Overall, our method provides a path towards a fully automated delineation of any type of tumor so long as there is a cancer-oriented molecular stain available for subsequent learning. Software, tutorials and interactive tools are available at:https://github.com/BiomedicalMachineLearning/HEMnet

## Background

Histopathological examination of tissue is indispensable for the accurate diagnosis and treatment of cancer^1–3^. Frequently, pathologic diagnosis of cancer and different subtypes dictate the use of specific treatment regimens^4^. One of the current standards of cancer diagnosis is microscopic examination of tumor tissue sections jointly stained with hematoxylin and eosin (H&E) dyes^2,3^. Based on the H&E-stained image of a biopsy section, pathologists can qualitatively assess cancer types, stages and estimates of tumor purity^3^. Furthermore, histopathologic examination frequently reports different types of cells, organic states, and/or cellular localization inside complex tissues^5^ although diagnosis concordance among pathologists remains low^6^. The visual inspection of histopathologic sections of biopsies is a time-consuming task and lacks quantitative measurements for cellular features^4^.

Recently, the emerging area of digital pathology has been developed as a way to digitize, store and distribute cancer whole-slide images (WSIs). This approach significantly improves the speed and access to cancer anatomical pathology. The increasing production of WSIs requires advanced computational approaches to be developed to analyze these medical images in a fast, robust and accurate manner, ultimately leading to applications in automated cancer diagnosis^7–10^.

Deep learning is the method of choice for analysis of digital histology images and many methods have been developed for tumor classification^8^. However, a key challenge for deep learning is the need for a very large number of accurately labeled data^11^. For this approach, many methods require WSIs that are manually annotated by a pathologist^12^. Thus, generating the training dataset becomes a time-consuming manual process, which still has the limitation in the high variation between pathologists^13^. This adds to the cost and makes it more expensive to obtain these training datasets^14^. Another challenge is that these slide images are large; an image at ×10 magnification can contain hundreds of millions of pixels. However, a pathologist’s annotations are often not at the pixel level and rely on much cruder methods of demarcation. As a result, training occurs at a lower image resolution that lacks cellular granularity^15^. We aim to address three key challenges, namely the dependence on the variable pathologist annotation for model training, the need to have a large number of images for training, and the demand to achieve a high-resolution and quantitative prediction of cancer cells.

Herein, we describe a new automated approach in which we use prior staining that demarcates tumors from normal cells at much higher image resolution. Immunohistochemistry (IHC) has been a useful tool in both research and clinical diagnosis—the classical histopathology method locates and visualizes specific cells or antigens based on antigen-antibody binding. Importantly, IHC is widely used for formalin fixed paraffin embedded (FFPE) tissue, the most common tissue archival method^16^. The manual coupling of H&E and molecular marker staining images for detection (by H&E) and further confirmation (by IHC) is increasingly being applied for histopathological diagnosis^6^. This also creates a valuable opportunity for digital data integration between tissue morphology and molecular profiles, an area that has not been utilized^2,17,18^.

We developed a method, referred to as H&E molecular neural network (HEMnet), which automatically aligns every pixel in the IHC image to the corresponding pixel in the same location on H&E image. Our approach labels each H&E pixel as biomarker positive or negative. For this proof-of-concept study, we used an IHC marker for cancer to delineate tumor cells. We used p53 staining, an important tumor suppressor gene (*TP53*), which is prone to a high frequency of genetic alterations across many different malignancies^19,20^. Most *TP53* mutations are of the missense class that change the p53 protein structure, making them more stable and has a much longer half-life than the wild-type form. *TP53* mutations result in the stabilization and subsequently accumulation of p53 in malignant cells^21^, allowing it to be readily detected by IHC. Wild-type p53 is unstable and has a short half-life, and thus p53 in normal cells is usually undetectable by IHC^22^. Up to 74% of colorectal cancer samples show abnormally high positive staining (i.e., a brown color) for p53, which provides specific IHC marker for cancer cells in colorectal cancer^19,20,23^. By mapping/registering p53 IHC image to H&E image, we improved the model training and testing dataset as described below.

Our study leveraged innovative molecular label transferring to generate tens of thousands of H&E tiles extracted from the WSIs, without manual inspection or with a minimal effort to confirm the automated labels. Here, HEMnet was trained on a set of p53-stained and H&E WSI images from colon cancer. We used aberrant p53 staining patterns to annotate colorectal cancer cells in H&E slides by aligning these images. With thousands of labeled tiles, a convolutional neural network classifier was trained based on an in-house colorectal cancer dataset. With this training and testing approach, we achieved a high performance on an independent set of histopathologic sections and images. HEMnet was extended to testing the Cancer Image Archive (TCIA), which has an extensive repository of colorectal cancer histopathology imaging data. By comparing with other genomics-based methods, we demonstrated a high performance with a significant positively correlation^24,25^. For generalization, so long as the molecular label is relatively specific to the tumor cells, this process should enable one to conduct streamlined and high-resolution molecular annotation of cancer versus normal cells. The HEMnet approach can be easily implemented with other interesting biomarkers such as HER2 and for other types of cancer. Recent developments of multiplex marker assays, like mass cytometry imaging, would enable label transferring of more than one markers to H&E images to allow for the analysis of cancer complexity to a greater extent. Given its success, this method has potential clinical application. One can use common histopathological images to enable the discovery of cancer cellular geometric patterns within the tissue and our software is capable of automatic detection of these patterns as part of developing computer aided diagnosis tool.

## Results

### Molecular information for H&E images annotation

**Fig. 1:**
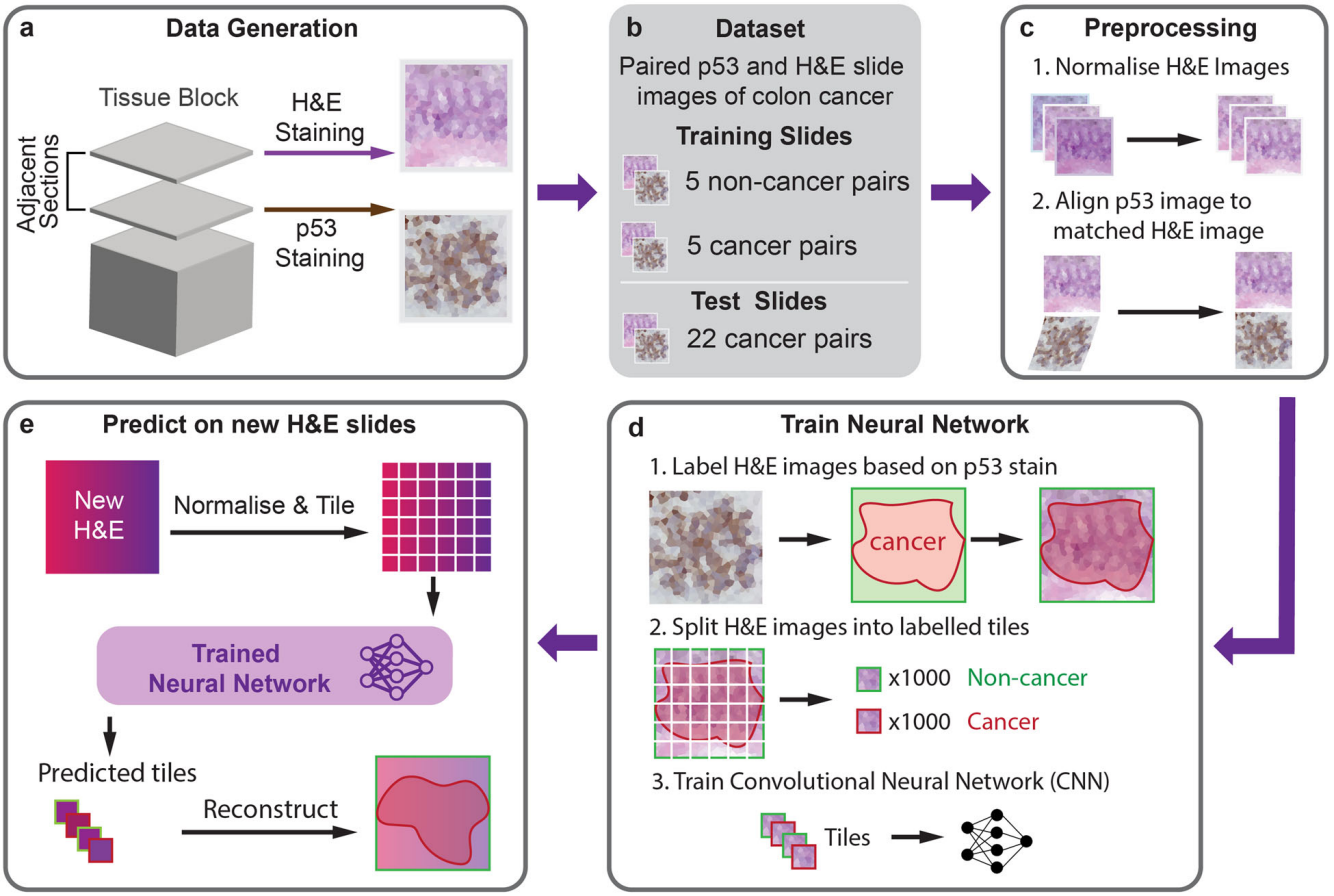
H&E Molecular neural network (HEMnet) workflow overview. **a** Matched p53 IHC stained and H&E-stained WSI derived from two adjacent tissue sections. **b** Training was performed on paired normal and cancer slides (five pairs). Test slides were held-back and are unseen by the model training. **c** Preprocessing to account for technical variations in slide preparation through stain normalization and image registration. **d** Molecular labels were transferred from p53 to H&E images. Post label transferring, each image was tiled to generate thousands of small samples (224 × 224 pixels) to train a CNN. **e** Application of HEMnet to predict cancer from new clinical H&E images.

We developed an approach which leverages molecular annotations and deep-learning methods to improve the identification of cancer cells (Fig. 1). The HEMnet development pipeline comprises four major steps: (1) data generation of paired P53 and H&E images, (2) preprocessing images and transferring of molecular label, (3) training neutral network, and (4) evaluating the performance of HEMnet (Fig. 1). The HEMnet pipeline was designed for applicability to any staining type or cancer type.

For this study, we developed HEMnet to identify tumor cells in H&E images of colorectal cancers. For step 1, we obtained 32 high-resolution H&E images and corresponding p53 IHC images from 27 cancer samples and 5 non-cancer samples. This was achieved by staining adjacent tissue sections with H&E and p53 to generate a matched paired WSIs for each tissue block. Step 2 is the novel contribution of HEMnet to transfer molecular labels to the H&E image. HEMnet takes advantage of molecular information, instead of manual pathologist annotations. We accomplished this by alignment of p53 molecular stained images to the corresponding H&E images at the pixel level (Fig. 3). The p53 stain pattern was, thereby, used to label cancer regions on the paired H&E images in an automated fashion, without the need for pathologist intervention. For step 3, each labeled H&E image was split into thousands of small tiles 224 × 224px so that from a small sample of 10 WSIs we can generate tens of thousands of training samples (Fig. 3d). We used these image tiles to train a deep-transfer-learning classifier to identify cancer regions in clinical H&E images using only tissue morphology features. Step 4 provides stringent validation criteria with independent datasets, comparing HEMnet with pathological annotation and with seven computational genomics methods.

### H&E stain normalization reduces color variation

**Fig. 2:**
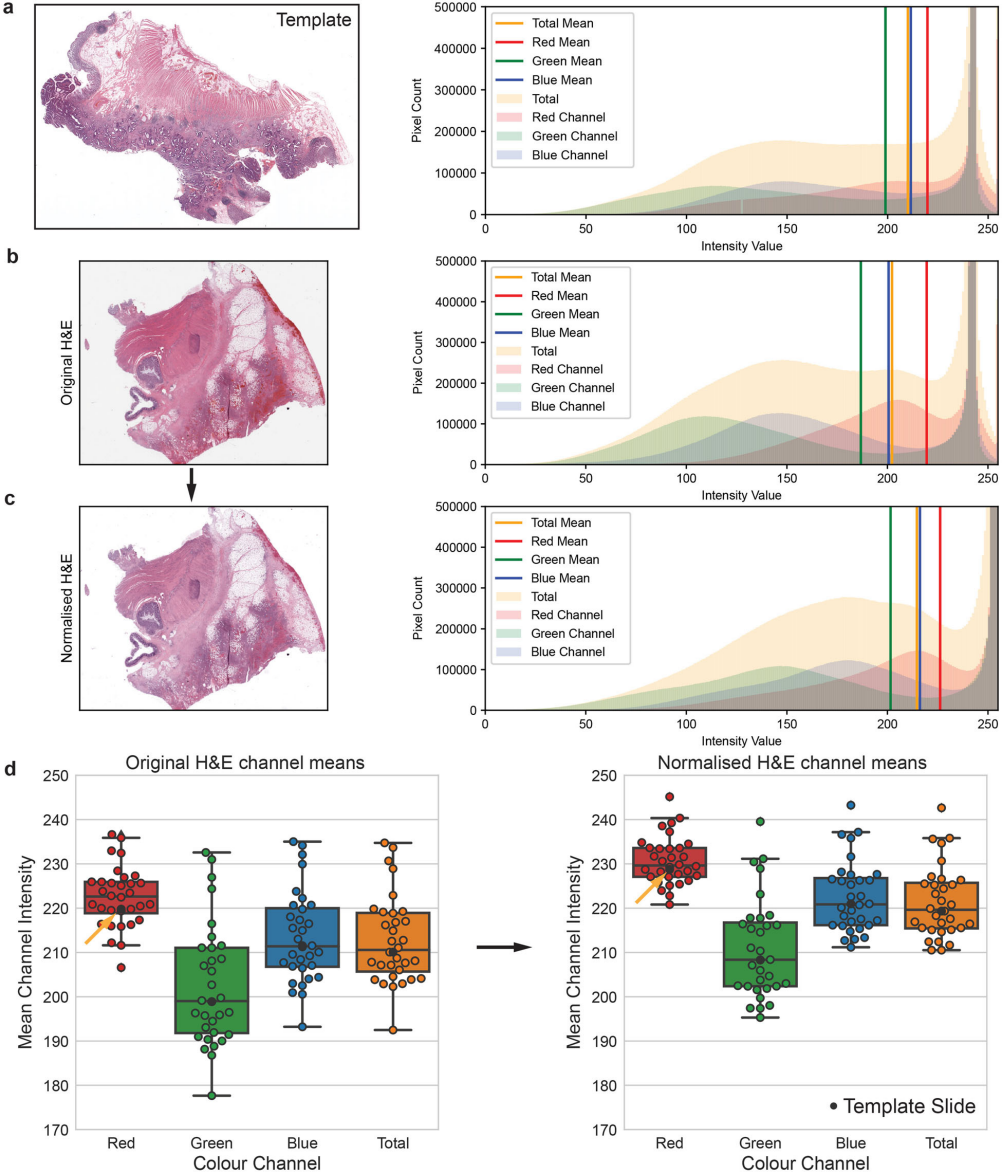
H&E stain normalization. **a** Template slide—cancer slide with mean R, G, and B channel intensities most similar to the median of the mean channel intensities of all images. Histograms shown for x2 magnification image (**a**, **b**, **c**). **b** H&E image before normalization. **c** H&E image after normalization more closely resembles template image. Image brightness is increased and pixel intensity distributions are retained. **d** Normalization of all slides (*n* = 32). Reduced variation of mean channel intensities after normalization was observed. Template slide means channel intensities are closer to the median (boxplot center line) after normalization (indicated by arrows) and interquartile range (boxplot bounding box) was shrunken. Boxplot whiskers indicate data range, excluding outliers.

Besides realizing the concept of using molecular labels in deep-learning model, the technical contribution of the HEMnet pipeline lies in the seamless pipeline, comprising a step to combine multiple images into a model training and testing dataset by normalizing different images, followed by fast and accurate label mapping, before training a neural network. Initially, WSIs with similar tissue structures stain different colors due to differences in slide processing (e.g., staining time, microscopy exposure). We address this issue with stain normalization, which caused these WSIs to take on the stain color profile of the template slide and increased the luminance to produce a white background (Fig. 2a–c and Fig. S2). This method changed the mean R, G and B channel intensities of the normalized slide to closely resemble the template slide whilst retaining the R, G and B color distributions within the image. Across the 32 H&E WSIs, stain normalization reduced the variation in mean R, G, and B channel intensities (Fig. 2d). In addition, it adjusted the median of the median channel intensities to move closer to the mean channel intensities of the template image. By normalizing all images before input into the model, we ensure the model can generalize to new slides stained differently to the training slides.

### Transferring p53 molecular labeling to corresponding H&E images

**Fig. 3:**
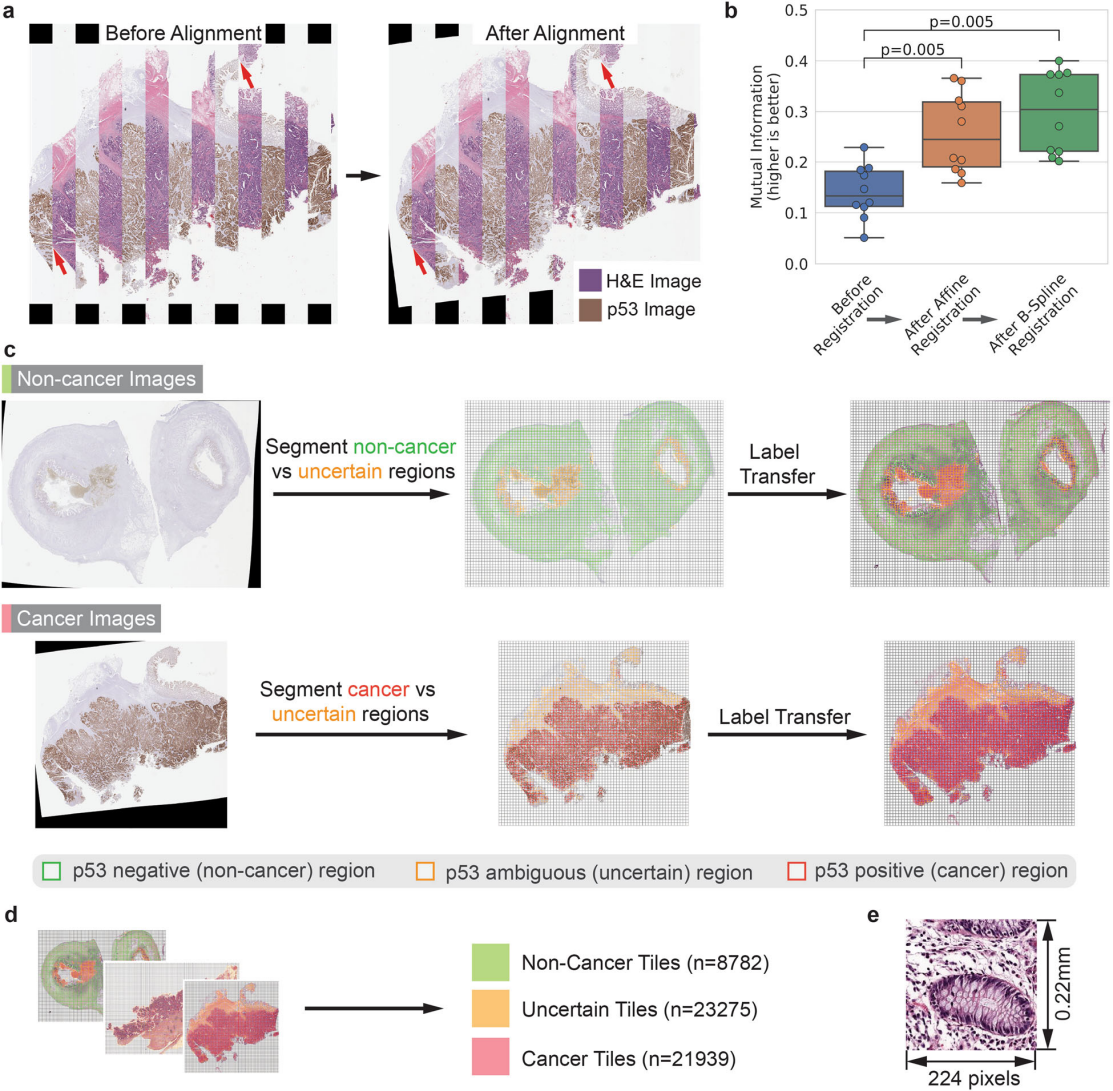
Molecular labeling of H&E images to train neural network. **a** Overlay of H&E and matched p53 image showing improved alignment after registration, highlighted by red arrows. **b** Accurate alignment of p53 images to corresponding H&E images. Successive affine and b-spline registration increases mutual information, a measure of image similarity. Significance testing with *t*-test. Boxplot center line indicates median value, bounding box shows interquartile range and whiskers, the data range. **c** Segmentation of p53 images to label matched H&E images where only non-cancer tiles are generated from non-cancer slides, vice versa. **d** 10 training H&E images generated tens of thousands of tiles, increasing sample size. **e** Example of cancer tile generated at ×10 magnification and used for training neural network.

The WSIs from corresponding p53 and H&E-stained slides often were misaligned (Fig. 3a). For the p53-positive cells to accurately map to cancer cells on the H&E images, we realigned p53 images to their corresponding H&E images though HEMnet automated image registration (Fig. 3c). Our intensity-based registration approach was fast and accurate as we optimized mutual information (Fig. 3b, c). Next, we labeled the H&E image based on the p53 staining pattern where p53-positive regions are labeled as cancer, vice versa. To counteract limitations of p53 staining in marking cancer cells, only p53-positive tiles from cancer slides and only p53-negative tiles from non-cancer slides were used for training. All the other tiles were labeled as uncertain and excluded from any additional processing. At ×10 magnification, a single WSI can generate thousands of tiles for training (Fig. 3c). We generated 224 × 224 pixel tiles from the molecular labeled H&E images to train a VGG16 deep-learning model (Fig. 3d).

### Molecular annotation quality-control produces a high-confidence dataset

The *TP53* tumor suppressor gene is the most commonly mutated gene in human cancers (50%) and disproportionately has mutations and other genetic alterations for up to 70%–80% of colon cancers^26,27^. As a result of its general prevalence, it provides a highly generalizable way to molecular annotate a broad range of cancers. Similar to other IHC markers, p53 staining has its limitations as within one image or between images, the marker is not always indicative of cancer, vice versa. For example, overexpression and positive staining for p53 may occur in normal cells responding to DNA damage. In addition, p53 may be absent in cancer cells with *TP53* gene deletions^22^. To overcome these limitations, when training our model, we only considered p53-positive cells as cancer if they come from a cancer slide and only p53-negative cells from slides where the cells have a normal morphology (Fig. 3d). In this way, we were confident that cells were correctly labeled, with 8782 non-cancer tiles and 21,939 cancer tiles. We removed 23,275 tiles that had some levels of uncertainty (Fig. 3d).

### High-performance automated assessment of cancer cell abundance and spatial distribution

**Fig. 4:**
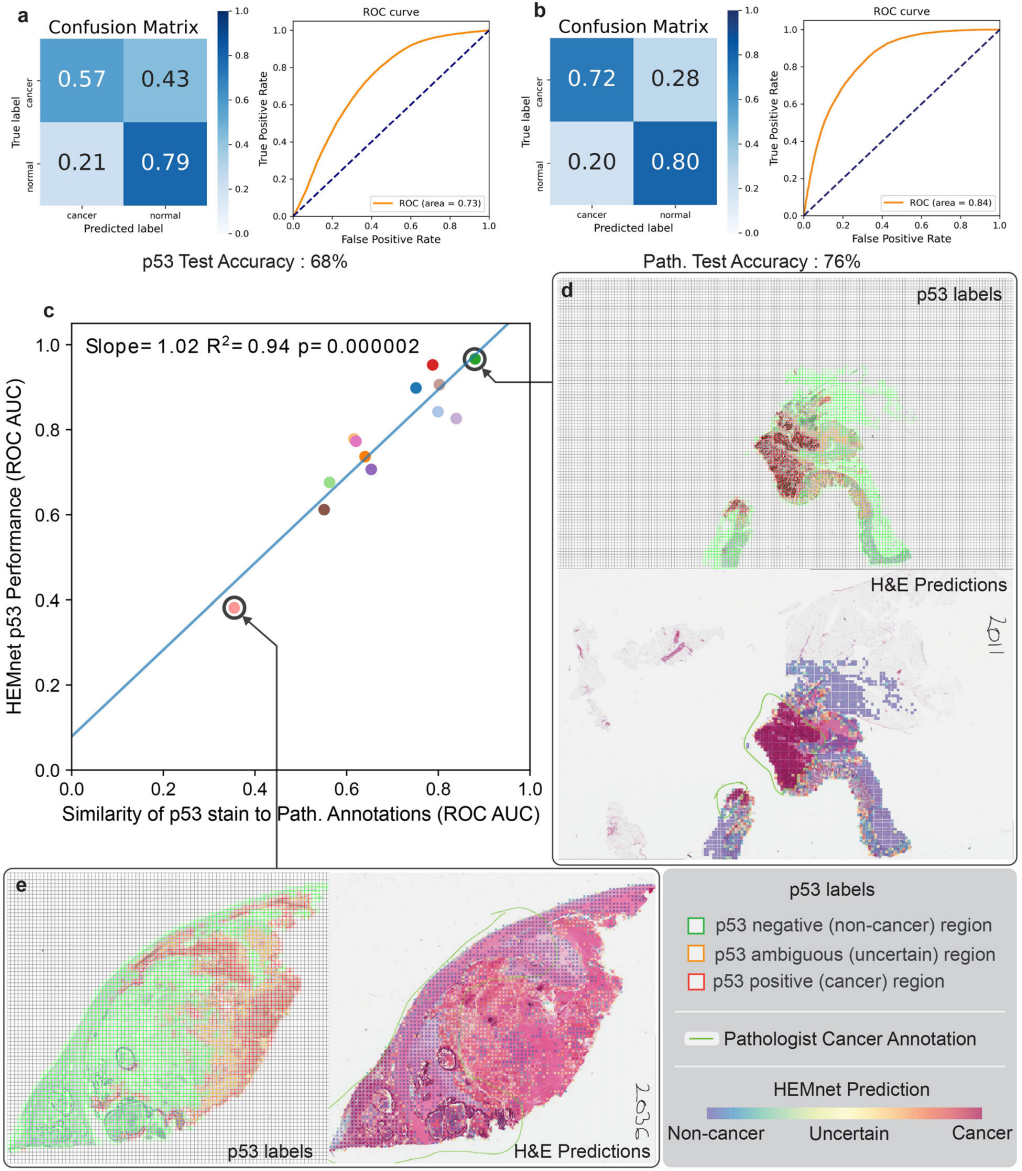
HEMnet performance on unseen H&E slides. **a** Prediction of p53 stain pattern on 17 unseen H&E slides. **b** Prediction of cancer regions on 13 unseen H&E slides compared to pathologist annotations. **c** Prediction performance of p53 stain pattern is positively correlated with the ability of p53 to mark cancer regions on the tissue, as annotated by pathologist. **d** HEMnet accurately predicts cancer regions annotated by pathologist (bottom) and p53 stain (top) when p53 stain agrees with pathologist annotation. **e** HEMnet predicts cancer regions even when p53 stain pattern (left) disagrees with pathologist ground truth annotations (right).

We applied the trained HEMnet to unseen WSIs to predict cancer regions. Of the 17 unseen H&E slides in the test dataset, all had corresponding p53-stained slides and 13 had additional pathologist annotation of the cancer region. We found that HEMnet could accurately predict p53 stain pattern (ROC AUC = 0.73) and pathologist annotated cancer regions (ROC AUC = 0.84), (Fig. 4a, b). These results suggest that p53-positive cancer regions for a given tissue sample can be predicted from its general morphology using a classifier developed with molecular labeled H&E images.

Comparing the p53 labeled tiles to pathologist labeled tiles from the same location, we found an overall agreement in tile labels (ROC AUC = 0.67) (Supplementary Fig. 6). However, this agreement was not absolutely perfect. To evaluate any discrepancies, for each slide we measured the ability of p53 stain to annotate cancer. This analysis involved calculating the ROC AUC between p53 stain and ground truth labels of tiles per a pathologist. We found that HEMnet p53 performance (ROC AUC) was higher in slides where p53 more accurately labeled cancer (p53 vs pathologist tile labels ROC AUC) with a significant correlation as noted by a Pearson coefficient of 1.02, and *R*^2^ = 0.94 (Fig. 4c). This result indicated that the model learnt to recognize specific morphology features of cancer cells and was not strictly limited to identifying cells with high levels of p53. This likely because cancer cells are morphologically distinct from normal cells whereas the differences in morphology between p53 positive and negative cells are more subtle. We noted that there were examples demonstrating that HEMnet can identify the cancer marked by the pathologist, even where the cancer is not identified by the p53 stain (Fig. 4d, e). Overall, the results suggest that HEMnet is able to accurately identify tissue morphology features of cancer.

### External validation and application to TCGA suggests the broad applicability

**Fig. 5:**
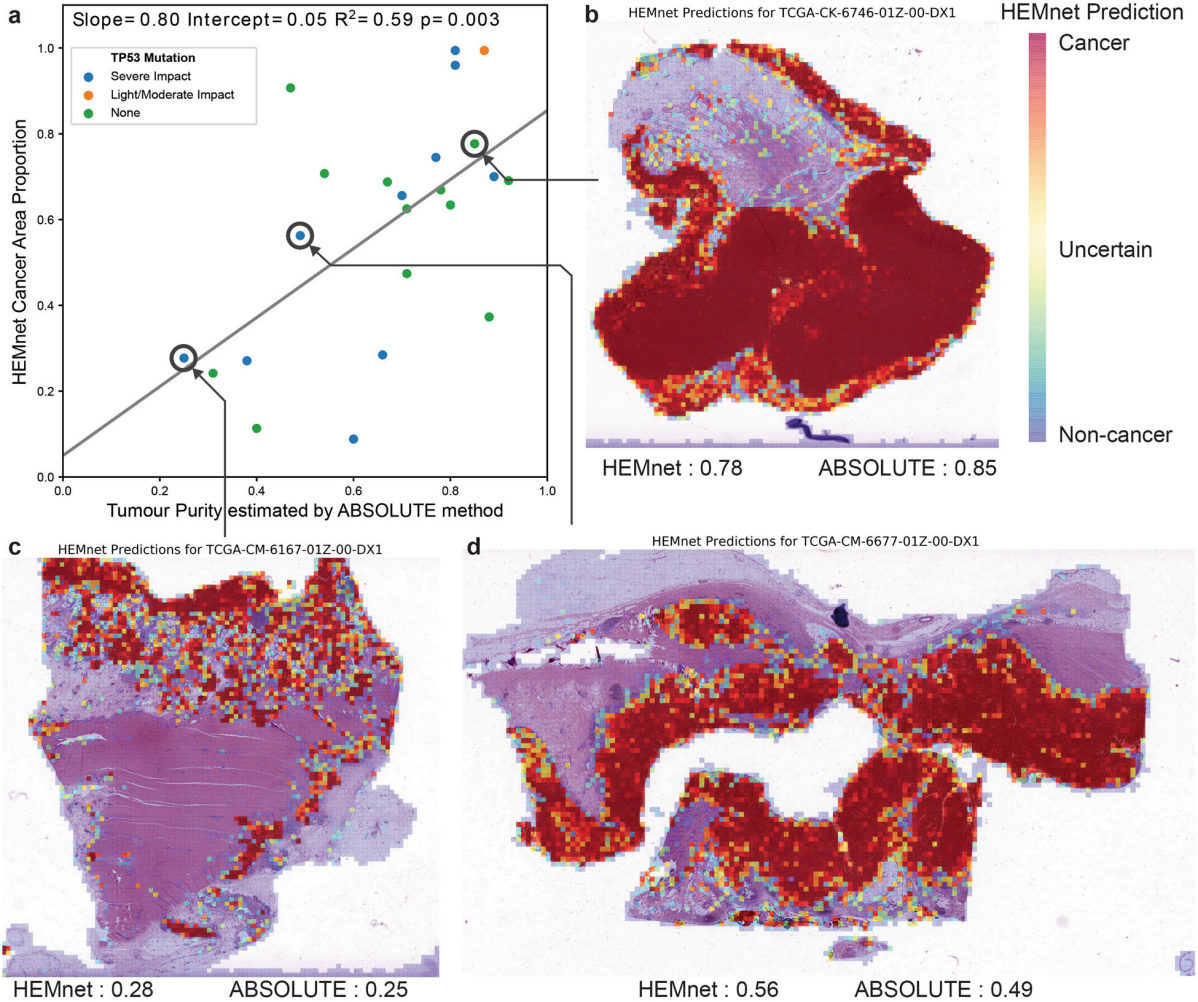
External Validation on The Cancer Genome Atlas (TCGA). **a** Comparison of HEMnet estimation of tumor purity—approximated by the proportion of cancer tissue area to total tissue area—to sequencing estimates of tumor purity using the ABSOLUTE method (*n* = 24). The colors of the dots represent three categories of TP53 mutations from the TCGA data. **b**, **c**, **d** HEMnet cancer predictions on formalin fixed TCGA slides for low (**c**), medium (**b**), and high (**d**) tumor purity colon adenocarcinoma.

As an independent validation using an external dataset, we applied HEMnet to colon adenocarcinoma samples from TCGA colon cancer samples. We used these CRCs to investigate the generalizability and clinical application of the method (Supplementary Table 2). The unmodified HEMnet model was trained by the in-house dataset described in this study to predict on H&E WSIs of colon adenocarcinoma. By combining the tile-level prediction with the cellular content of each tile, we calculated the proportion of cancer tissue to total tissue for each slide (Supplementary Table 2 and Fig. 5a). This acts an approximation of tumor purity which we compared to sequencing method estimates from matched genomic data. There are several differences between our colon cancer data and the TCGA data. Most importantly, the sequencing was not performed on the same tissue used for diagnostic imaging. Despite these challenges, we found a significant correlation between our method and tumor purity as estimated by ABSOLUTE, with a regression coefficient of 0.8, as shown in Fig. 5. Furthermore, we examined if the performance of HEMNet is affected by the following factors; (i) TP53 mutations status, (ii) clinical stages, (iii) MSI status, and (iv) CMS-RF classifier. We found that HEMnet performs well regardless of the TP53 mutation background (Fig. 5a and Supplementary Fig. 8). Other factors did not affect the performance of HEMnet significantly. These results suggest that HEMnet can generalize to new colorectal clinical data and is able to reliably predict on TCGA images. As we observed, our prediction is accurate in general for detecting true positive (cancer cells) and true negative (normal cells), but it also has small tissue proportion with false positives (predicting normal epithelia cells as cancer cells, often found as ambiguous regions with HEMnet probability scores lower than those for cancer regions). However, we believe that the tiles annotated with our prediction scores could assist a pathologist to examine slides quickly and validate ambiguous areas (Supplementary Fig. 9).

## Discussion

Histopathological examination of H&E images has been the gold standard for pathologic diagnosis of almost all suspected cancer patients^3,28^. Modern applications of machine-learning tools to analyze H&E images are increasing being used recently^7,29^, with some of the computer-assisted image diagnosis tools already approved by the Food and Drug Administration (FDA)^30^. Hundreds of deep-learning methods have been developed to use just H&E images to detect and diagnose cancer^7^. Although some of these methods have achieved high performance, they all rely on pathological annotation for labeling/segmenting images into multiple tissue regional classes^7,31^. Notably, the gold-standard annotation by pathologists is not always the ground truth and there is inherent variation in annotation between pathologists. In the case of melanoma, for example, the intra-observer reproducibility was low, for class II (35.2%), class III (59.5%), and class IV (63.2%)^32^. Most methods also require a large number of annotated images for model training and evaluation^33,34^ and the lack of large annotated datasets is a major challenge for deep-learning image analysis^7^. We developed HEMnet as a cancer diagnosis framework that uses digital labeling and neural network to address these challenges.

HEMnet combines two common types of histopathological WSI data, namely H&E staining and immunohistochemistry staining images. The novelty in HEMnet pipeline lies in the molecular label transferring, which allows for the use of pixel-level molecular information cancer cells (e.g., P53 positive/negative pixel) with thousands time higher resolution than manual pathological segmentation. In HEMnet, we solved several key technical challenges to allow for accurate, fast and generalizable label transferring, with the ultimate aim that HEMnet can be implementable to different datasets, including those with a high level of technical variation. Briefly, technical variation is introduced by the tissue sectioning, mounting, staining and imaging processes. Very few studies investigated the intrinsic technical variations, like contrast, brightness, or signal to noise^7^. Different to most methods, HEMnet implements an optimized pipeline for preprocessing, allowing removal of technical variation between images. HEMnet include functionalities to thoroughly perform background correction, normalization, alignment, registration, and label transferring. Prior to normalization, luminosity standardization was performed to correct for image brightness. We compared three normalization methods, Vahadane^35^, Reinhard^36^ and Macenko^37^, and confirmed the better performance of the Vahadane method (set as default). The image registration implements a probabilistic approach with mutual information maximization. We compared multiple options and found that intensity-based registration, and the sequential combination of Affine followed by B-spline registration^38^, using a gradient-descent-based optimizer to minimize mutual information loss perform well for registering H&E image data. We also assessed the computation and running time, as registration is an intensive process. Down-scaling was found as a practical solution. Finally, to label the registered image, we developed a tile-level thresholding strategy to distinguish cancer, non-cancer and uncertain labels for every tile of 224 * 224px. The tile-level labeling with thresholding, categorizing and filtering steps allows us to create a high-quality training (and evaluation) dataset for neural network, minimizing the technical noise from registration errors and uncertain labeling.

Overall, the label-transferring solution implemented in HEMnet represents a significant technical advance and is needed to the increasingly important digital histopathological analysis field. The label transferring brings about three key beneficial effects on model training. First, the pixel-level labels allow us to divide one image into hundreds to thousands of smaller, high-resolution, molecular labeled tiles, thereby increasing sample sizes for model training and testing. This enables development of accurate models with few slides, unlike existing methods which require a thousands of WSIs^7,33^. In general, tiling of WSI yields the large amount of data for training neural network and thus is able to overcome gaps in image assignment. This feature was demonstrated by the fact that HEMnet successfully identified some non p53-stained cells as cancer cells (Fig. 4). With pixel-level labeling, the classification of cancer cells is at hundreds to thousands of times higher resolution than macroscopic drawings by pathologists. Moreover, molecular labeling is automated, making the output less dependent on the laborious, manual and variable annotations by trained pathologists.

HEMnet, with its label transferring approaches, can be beneficial for a large range of applications. When processing an independent validation set not used in the original-learning process, HEMnet predicted the same overlapping region delineated though a pathology annotation (ROC AUC = 0.84). We validated HEMnet by systematically comparing HEMnet with other methods and with the ground truth pathological annotation. We found highly correlated results with other independent methods (correlation coefficient in predicting cancer purity = 0.8) using TCGA dataset^39^.

We selected p53 staining, an established marker for cancer cells, to develop HEMnet label transferring as we expected that well studied problem allows us to evaluate the performance of our algorithm. Among non-cancer cells, p53 protein is usually undetectable by IHC^22^, whereas up to 74% of colorectal cancer cells stained positive for p53 with brown color^19,20,23^. The generalization to other types of markers and cancer, for example HER2 for breast cancer, is possible with further validation. The feasibility of correlating H&E images with IHC images by deep neural networks has been investigated for the case of SOX10 staining^40^ and fluorescent cancer marker images like pan-cytokeratin (panCK), or α-smooth muscle actin (α-SMA)^41^. HEMnet was developed using p53 IHC staining as an appropriate colorectal cancer marker that is expressed in 70%-80% of colon cancers^19^. We expect that the HEMnet label transferring and thresholding approaches to define positive cancer labels can be generalized to other cancer types and immunohistochemistry markers. HEMnet can be readily adapted for training on new data—the analysis framework takes into account technical variation and scalability as discussed above. We confirmed by the test on the TCGA dataset robust performance. The label transferring pipeline can be expanded to many other applications to integrate imaging data from adjacent tissue sections. We made HEMnet an easily adaptable tool for most users through the interactive Google Colaboratory workspace, which allows users to upload their data and use our pretrained model for neural network prediction.

In conclusion, HEMnet is currently the unique molecular modeling approach that utilizes both H&E and IHC images for quantitatively classifying cancer cells within tissue sections. We expect that HEMnet has the potential to be used as a computer-assisted tool that help pathologists by suggesting important regions, such as cancer parts, in the tissue^29,42^. HEMnet does not require human pathological annotation, automatically labeling images at pixel resolution. The application of software like HEMnet can benefit cancer diagnosis by unprecedented resolution, efficiency, reproducibility, accuracy, speed, reduced cost and increased access to pathological services. In an aging society where more biopsies are available while there is a lack of professional anatomic pathologists^43^, such computational innovation is increasingly important. We believe HEMnet can further accelerate computational pathology application and integration into the pathology workflow routine, assisting in disease diagnosis and ultimately removing missed diagnosis and improving patient outcomes. We provide HEMnet as an open-source software and also as an accessible cloud-based prediction tool that allow users to analyze their images without a requirement for further programming.

## Methods

### H&E and IHC image dataset generation

We collected cancer tissue samples from 30 patients at Stanford Hospital. All patients were enrolled according to a study protocol approved by the Stanford University School of Medicine Institutional Review Board (IRB-11886). All participants provided written informed consent to take part in the study. Tissues were obtained from the Stanford Cancer Institute Tissue Bank. In addition, we obtained matched normal, non-cancer tissue from five patients. Each sample was formalin fixed and paraffin embedded (FFPE) as a tissue block and two adjacent sections were taken from each block, ensuring these sections would close to identical. One section was prepared with H&E staining and the other with IHC staining against p53 using DO-7 monoclonal antibody (Roche, Cat# 790–2912, prediluted) by Anatomic Pathology & Clinical Laboratories at Stanford Medicine. All digital slide images were generated in Aperio SVS format by Translational Pathology Core Laboratory at University of California, Los Angeles. This study was conducted in compliance with the Helsinki Declaration. Each tissue section was scanned at ×20 magnification to generate a total of 35 of p53 and H&E pairs of high-resolution WSIs.

### Training, validating, and testing dataset generation

We use a common practice in machine learning of splitting our dataset of WSIs into training, validation and test sets. No overlap existed between these datasets to ensure that test and validation data was completely independent. We assigned the five normal WSI pairs and five cancer WSI pairs to the training dataset. To ensure an accurate training dataset, we also confirmed that most p53-stained regions were cancer in these slides by a pathologist. Altogether, this provided the model the optimal degree of learning to distinguish between cancer and non-cancer tissue (Supplementary Fig. 1a). The WSIs were captured at gigapixel scale (Supplementary Fig. 1b) allowing us to employ a tiling strategy to split each WSI into thousands of smaller 224 × 224px image tiles for neural network training. We set aside five cancer WSI pairs as a validation dataset to optimize our model’s hyperparameters. The remaining 17 cancer WSIs were assigned to an independent test dataset to assess our model’s performance on unseen slides.

### H&E stain color normalization

Undesirable color variations occur in H&E staining and imaging due to different immunohistochemistry reagents, protocols and slide scanners^35^. Therefore, the same cellular structures in a tissue can appear different depending on how the tissue was stained and imaged. To ensure our model generalized to images from H&E slides across different facilities, we corrected for technical variations in the staining and imaging process. First, we corrected for imaging brightness and ensured that the slide background is white through luminosity standardization (Supplementary Fig. 2). Next, we normalized each H&E WSI to a reference stain color profile derived from a template WSI using the Vahadane et al^35^. stain normalization method implemented in StainTools^44^, Eq. (1).

1 $${OD}_{{flat}} = C \ast S$$

The OD_flat_ is the flattened optical density (OD) array derived from the RGB WSI. A stain matrix (*S*) encodes the stain color for the H&E staining and is estimated using the Vahadane method. This stain matrix is used to find the pixel stain concentration matrix (*C*). To normalize a source WSI to a template WSI, the stain and concentration matrix for both images are calculated, as per Eqs. (2) and (3).

2 $${OD}_{{source}} = C_{{source}} \ast S_{{source}}$$

3 $${OD}_{{template}} = C_{{template}} \ast S_{{template}}$$

The *C*_source_ matrix describes the concentration of hematoxylin and eosin stain at each pixel. Using the stain matrix from the template image (*S*_template_) we colored each pixel in source concentration matrix to produce an image (Eq. (4)), as if the source image was stained and captured the same way as the template image.

4 $${OD}_{{norm}} = C_{{source}} \ast S_{{template}}$$

By normalizing all WSIs, training and unseen, to the template image, we ensured that similar cellular structures have the similar appearances regardless of how they were stained and underwent image scanning.

To select a suitable template WSI, we find the cancer slide with mean R, G, B channel intensities closest to the median of the mean of the different channel (R, G and B) intensities of all images (Supplementary Fig. 1c). In addition, we implemented two user-selectable, popular but less advanced, image normalization methods by Reinhard et al.^36^ and Macenko et al.^37^.

### Registration of IHC images to H&E images

For the IHC images to be used to accurately label the H&E images, each IHC image was aligned with its corresponding H&E image. Despite originating from adjacent sections of the same tissue block, technical differences in sectioning, mounting and imaging caused misalignment between IHC images and their H&E counterparts. We aligned these images by implementing image registration through the SimpleITK package^38^.

During registration, the IHC images were warped such that they were aligned to the H&E images. By only transforming the IHC images we ensured that the H&E images remained unaltered. Technical variation among H&E images, for example the variation in the brightness, or color intensities due to microscopy exposure time and/or staining time, was normalized (Supplementary Fig. 2 and Fig. 2). Thus, a neural network trained on these H&E images can be applied to new normalized, but otherwise unmodified, H&E images.

We verified the accurate registration through visual inspection and a quantitative mutual information metric. We overlaid the registered p53 over the corresponding H&E image to visually check for correct alignment. In addition, we compared the alignment of p53 image to the H&E image by computing the mutual information between these images before, during and after registration. Mutual information is an information theory concept that can be applied to measure image registration performance (Supplementary Fig. 3). An increase in mutual information after registration is indicative of a better image alignment. The mutual information between the IHC and H&E image can be calculated using Eq. (5).

5 $$I\left( {{IHC},{H}\& {E}} \right) = \mathop {\sum }\limits_{{ihc},{h}\& {e}} p({ihc},{h}\& {e}){log}(\frac{{p({ihc},{h}\& {e})}}{{p({ihc})p({h}\& {e})}})$$

where *p*(ihc) and the *p*(h&e) are the marginal probability distributions of grayscale pixel intensities in the IHC and H&E image, respectively. The *p*(ihc, h&e) is the joint distribution of the images’ grayscale pixel intensities.

Registration strategies can broadly be segregated into feature-based and intensity-based methods. Feature-based methods extract features (e.g., corners) or fiducials from the source and target image and transform the source image such that features in the source image are in the same location as matching features in the target image. On the other hand, intensity-based methods consider the pixel intensity or intensity distributions. These methods also transform the source image such that it most closely correlates with the pixel intensities or intensity distributions of the target image, as measured by a cost function. In preliminary testing, we found that an intensity-based approach was effective for H&E images.

For our intensity-based registration approach, we selected a mutual information cost function to quantify the extent of registering the source and target images. This cost function measures the mutual information between the pixel intensity distributions of the source and target image. The goal of registration is to transform the source image such that the mutual information between the source and target image is maximized—this would imply a well registered image. The mutual information is calculated from grayscale pixel intensities so the IHC and H&E-stained images were first converted to grayscale. Post-registration, the optimal transform for the grayscale IHC image is applied to each channel of the RGB IHC image to produce a registered color image.

To achieve accurate registration and reach a global, rather than local optima, we performed affine registration followed by b-spline registration. The initial linear affine registration is limited to translation, scale, shear and rotation transformations whereas the subsequent b-spline registration is a non-linear transformation. The initial affine step ensures that large architectural features in the image are registered before b-spline registers the finer cellular features. The affine and b-spline transformations are both tuned by a gradient-descent-based optimizer to minimize the mutual information cost function.

Each affine and b-spline registration step incorporates a multi-resolution approach. The concept here is similar; to achieve better registration by registering large features before small features. At the beginning of the affine and b-spline step, a low-resolution image is used to encourage registration of the large features in the image. Gradually higher and higher resolutions are used to register every so finer features until the desired final resolution is reached. As registration is a computationally intensive process, especially for gigapixel WSIs, we registered smaller versions of the IHC and H&E images that were downscaled by 5 times - the downscale factor is user-adjustable. The final output of registration was color 5x downscaled IHC images accurately registered to corresponding H&E images of identical size. As the H&E images may have captured a different field of view compared to the IHC images, any out of image pixels in the IHC images were filled in with white.

### Automated labeling of images based on p53 staining

Registration transformed the p53 image to the same coordinate system as the corresponding H&E image. Thus, every pixel in the aligned p53 image referred to a pixel in the same location on the corresponding H&E image. This alignment was crucial for the p53 stain to accurately label the H&E image.

To label each pixel as one overlapping with cancer versus normal tissue, we applied thresholding to the p53 image. This process determined which pixels were positively (cancer) or negatively (normal) stained. The p53 IHC stain was visualized by the deposition of DAB (3,3′-Diaminobenzidine) on the tissue, giving positively stained tissue a brown color. We distinguished DAB-positive pixels, and hence p53-positive pixels, from the rest of the image by deconvoluting the RGB image into separate hematoxylin, eosin and DAB channels. This process was based on a method developed by Ruifrok and Johnston^45^. In this way, we could focus our thresholding on the DAB stain, which reflects the level of p53 protein at each pixel.

We observed that the pixels within the DAB channel fell into three classes: p53-positive pixels; faint tissue background staining which we interpret as p53-negative staining; pixels of slide background where there is no tissue and no p53 stain. To simplify this into a two-class thresholding problem, we used the hematoxylin channel to separate the tissue from the slide background—we applied separate thresholding to the tissue only regions of the DAB channel. In both cases, we used Ostu thresholding which maximized the inter-class variance between two classes. Through segmenting the tissue with the hematoxylin channel, we distinguished the tissue by its low, but considerably greater than slide background, levels of stain. In addition, it ensured that we retained the nuclei which have high levels of hematoxylin and is where the p53 protein is localized. Following tissue thresholding, we applied the Otsu thresholding to only the tissue regions of the DAB channel and separated each pixel into two classes: a p53-positive class of high intensity pixels; a p53-negative class of low intensity background-stained pixels. This process was applied automatically and independently to each p53 slide so that pixel misclassification did not occur because of subtle differences in staining between p53 slides.

We split each H&E image into 224 × 224px tiles for model training and testing. Subsequently, we translated p53 pixel level classification to tile-level cancer/normal classification. The registered p53 image was 5x down sampled to facilitate registration and it was on this image that we determined pixel and tile labels, as it is aligned to the H&E. Thus, we analysed and labeled 5x down sampled tiles of 45px × 45px, of equivalent field-of-view to the original image. These tiles contain multiple cells—within a tumor infiltrated region of tissue, not all of these cells will be cancer. To ensure that we did not miss cancer cells while minimizing the levels of false staining, we labeled a tile cancer if more than 2% of the pixels within the tile were p53 positive. The remaining tissue tiles were labeled as normal or’non-cancer’.

### Additional strategies to ensure accurate tile labeling

Pathology review provided the cancer versus normal cell status of these tissues. Three samples stained positive for p53 despite no histopathologic indications of tumor cells, which would have led to inaccurate labeling and model misclassification. To ensure accurate model training and testing, the p53 and H&E WSIs from these samples were excluded in the analysis. Overall, this left a total of 32 pairs of H&E and p53 WSIs, 27 cancer and five normal tissues.

In some cases, the p53 stain was not distinct enough to provide a definitive label to a tile so we labeledc ambiguous tiles as uncertain and discard them. These ambiguous tiles may add noise to the training data and prevent accurate evaluation of the model’s performance. We addressed this issue by setting an upper and lower user-selectable DAB intensity thresholds to enable labeling of tiles as uncertain. These thresholds were applied to the mean DAB intensity of each tile. Tiles that that fell between these thresholds were labeled as uncertain and were not used for training or testing the model. The remaining cancer and non-tumor tile labels were transferred from the registered p53 image to the H&E tiles destined for model training.

To safeguard against any registration errors and ensure accurate label transfer, if a p53/H&E pair of tiles had only one tile containing tissue, that H&E tile was discarded. To assess a tile, we segmented the tissue from the background in both p53 and H&E images using the GrabCut algorithm by Rother et al.^46^. In addition, to ensure a clean training dataset, only cancer-positive tiles from cancer samples were used and only cancer-negative tiles from the non-cancer samples were used.

### Training a convolutional neural network (CNN)

We trained the model with 224 × 224px tiles from ten H&E WSIs at ×10 magnification. Owing to our tiling strategy, we could generate thousands of samples from each WSI which we pooled together for training the model. We used transfer learning to develop a VGG16-based CNN for classifying tiles as cancer or non-cancer. Our model utilized a VGG16 architecture and was pretrained on ~1.3 million images from ImageNet^47^, for feature extraction. HEMnet has multiple options to implement CNN models during the image training, including ResNet50, VGG16, VGG19, InceptionV3, and Xception. We compared these models and found similar performance, with VGG16 running slightly faster and producing a higher accuracy (Supplementary Table 1). In fact, our HEMnet-VGG16 model has much fewer (>1000 times) parameters than in the original VGG16 model (Supplementary Fig. 4) because we only used VGG16 feature extractor with a transfer-learning approach where the parameters in the CNN base model are not trained. In addition, the max pooling layer (1,1, 512) output from this pretrained model was used as input to train a fully connected layer of 256 neurons), which output one sigmoid neuron with class probability for TP53 binary label. By using weights pretrained on a large number of images, we can train our model a relatively small dataset and still achieve accurate predictions without overfitting. Features from each 224 × 224px tile were fed into a fully connected neural network to predict tile cancer status.

The complete CNN was trained on labeled H&E tiles generated from the 10 training WSIs at ×10 magnification, for 100 epochs. We employed data augmentation to overcome overfitting and improve model generalizability. Since a given tissues extent of tumor cell infiltration remains the same regardless of the viewing angle or orientation, we randomly rotated and flipped tiles. The hyperparameters that performed best on the validation set were used for training the model that was used on all testing of unseen slides in this work. We implemented this system with Python using Tensorflow as the deep-learning framework.

### Performance evaluations

We tested our model on H&E test slides, evaluating its performance compared to p53 stain patterns and pathologist annotations. We measured model performance by computing accuracy, confusion matrices and receiver-operating curves (ROC). To evaluate performance against p53 annotations, we generated a test dataset using the same method described for the training dataset. Given that the sections had cellular mixtures, we generated tiles that solely represented cancer and normal tissues. For 13 of the 17 slides, we acquired pathologist cancer annotation drawings on the WSIs. We extracted the annotations and labeled tiles enclosed by the cancer annotation as cancer and labeled the remaining tissue tiles as non-cancer (Supplementary Fig. 5).

The main performance metrics are accuracy and ROC AUC. These are calculated by comparing the p53 and pathologist test dataset tiles labels with the labels predicted by our model **(**Figs. 4, 5 and Supplementary Fig. 6). Since cancer and non-cancer tiles do not evenly distribute in these datasets, we balanced the number of tiles for each class by subsampling the dominant class.

### TCGA validation

We validated our model on 24 colorectal cancer with H&E images. The WSIs were obtained from the TCIA and matched genomic data was retrieved from The Cancer Genomic Atlas (TCGA). The TCIA and TCGA are public repositories of cancer medical imaging data (including digital histopathology data) and cancer genomic data, respectively. We used our model predictions to estimate tumor purity and compared this to estimates of tumor purity derived from genome sequencing studies. For this image-based analysis, we calculated the proportion of the cancer tissue area to total tissue area by weighting tile predictions by the area of tissue within each tile. This is more accurate than using the proportion of cancer tiles to all tiles as some tiles, especially on the edge of the tissue. For example, a tile that is half background and half tissue would only contribute half a tile worth of area. We compare our estimate to seven method for determining tumor purity. This comparison included the programs ABSOLUTE^48^, EXPANDS^49^, ESTIMATE^50^, CPE^51^, InfiniumPurify^52^, and LUMP (leukocytes unmethylation for purity) (Supplementary Fig. 7).

### Reporting summary

Further information on research design is available in the Nature Research Reporting Summary linked to this article.

## Supplementary information

All supplemental figures and tables

REPORTING SUMMARY

## Data Availability

The datasets (all high-resolution H&E and TP53 images) used and/or analysed during the current study are freely available from the https://dna-discovery.stanford.edu/research/web-resources/HEMnet. The results for ABSOLUTE, ESTIMATE, CPE, InfiniumPurify, LUMP used for comparison with HEMnet were from “Supplementary Data 1” available at 10.1038/ncomms9971.

## Code availability

The source code, tutorials, and interactive analysis tools are available at https://github.com/BiomedicalMachineLearning/HEMnet. We also provide cloud-based implementation of the HEMnet (Supplementary Fig. 10), available as Google Colab notebook and an ImJoy application (links to these apps are on HEMnet github page). HEMnet is also available as an open-source PyPI python package (https://pypi.org/project/hemnet). The HEMnet software version 1.0.0 was used and the version information of software dependencies are listed in the HEMnet github site in the environment.yml file. We used SimpleITK version 1.2.3 for image registration and Staintools version 2.1.2 for normalization. EXPANDS 2.0.0 was applied for estimating tumor purity using the default setting.
