# Supplementary material for: A deep learning model for molecular label transfer that enables cancer cell identification from histopathology images
Source: NPJ Precis Oncol. 2022 Mar 2;6:14. doi: 10.1038/s41698-022-00252-0 (PMC8891271; doi:10.1038/s41698-022-00252-0)
Supplement: Supplementary file 1 — All supplemental figures and tables [file 41698_2022_252_MOESM1_ESM.pdf]

SUPPLEMENTARY INFORMATION

Supplemental Figures:

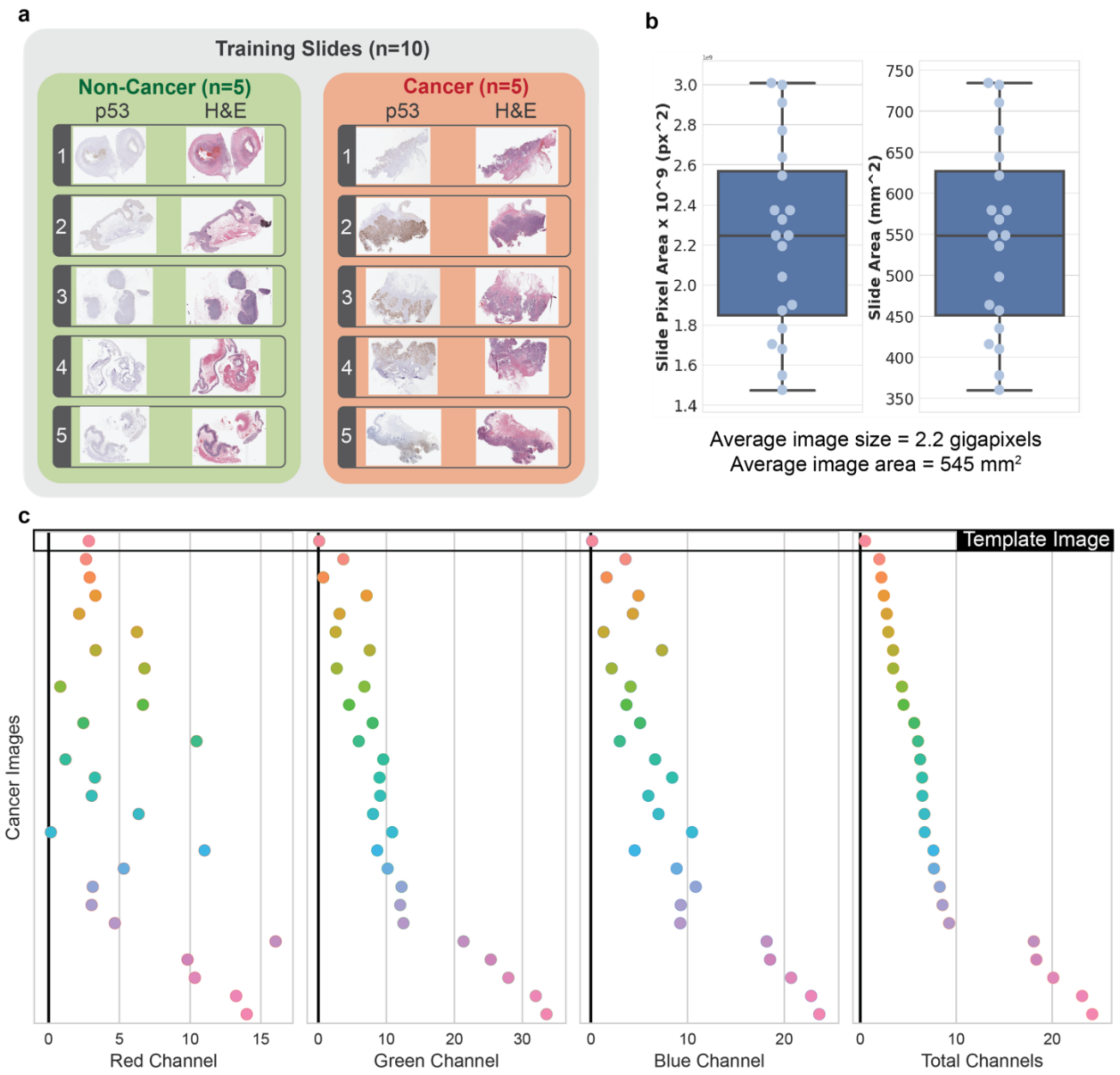

Supplemental Figure 1. **Overview of Dataset. a,** Training slides containing paired H&E and p53 slides – 5 pairs cancer, 5 pairs non-cancer. **b,** Distribution of training slide pixel areas in gigapixels and slide areas in mm^2. Boxplot centre line indicates median value, bounding box shows interquartile range and whiskers, the data range. **c,** Comparison of the mean R, G, B and total channel intensities of cancer images with the median channel intensities of all images. Each subplot shows the absolute difference between the mean intensities of cancer images to the median intensity of all images, for a particular channel. The cancer image most similar to the median of all images is selected as the template image against which all other images will be normalized against.

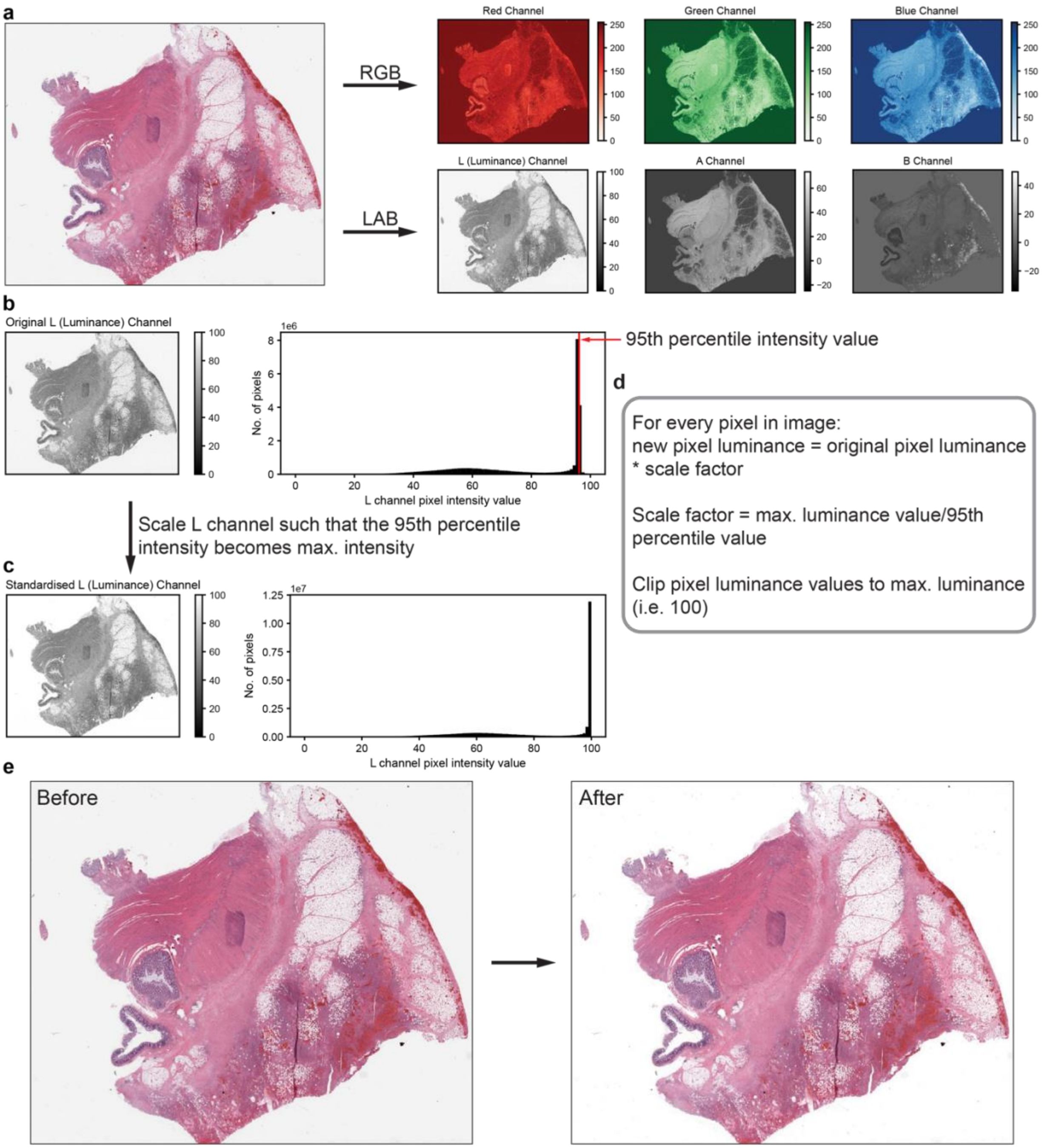

Supplemental Figure 2. **Luminance Standardization workflow. a,** Conversion of H&E WSI from RGB colour space to LAB colour space. **b,** Luminance (L) channel of WSI shows that few pixels are at 100 intensity, indicating the background is gray and not white. Histogram shown for 2x mag. image (**b, c**). **c,** Scaling of luminance (L) channel intensity shows that 5% of the slide is at max luminance – this makes the background white. **d,** Pseudocode concept of luminance standardization as applied to each pixel. **e,** Before and after standardization showing transformation of background from gray to white.

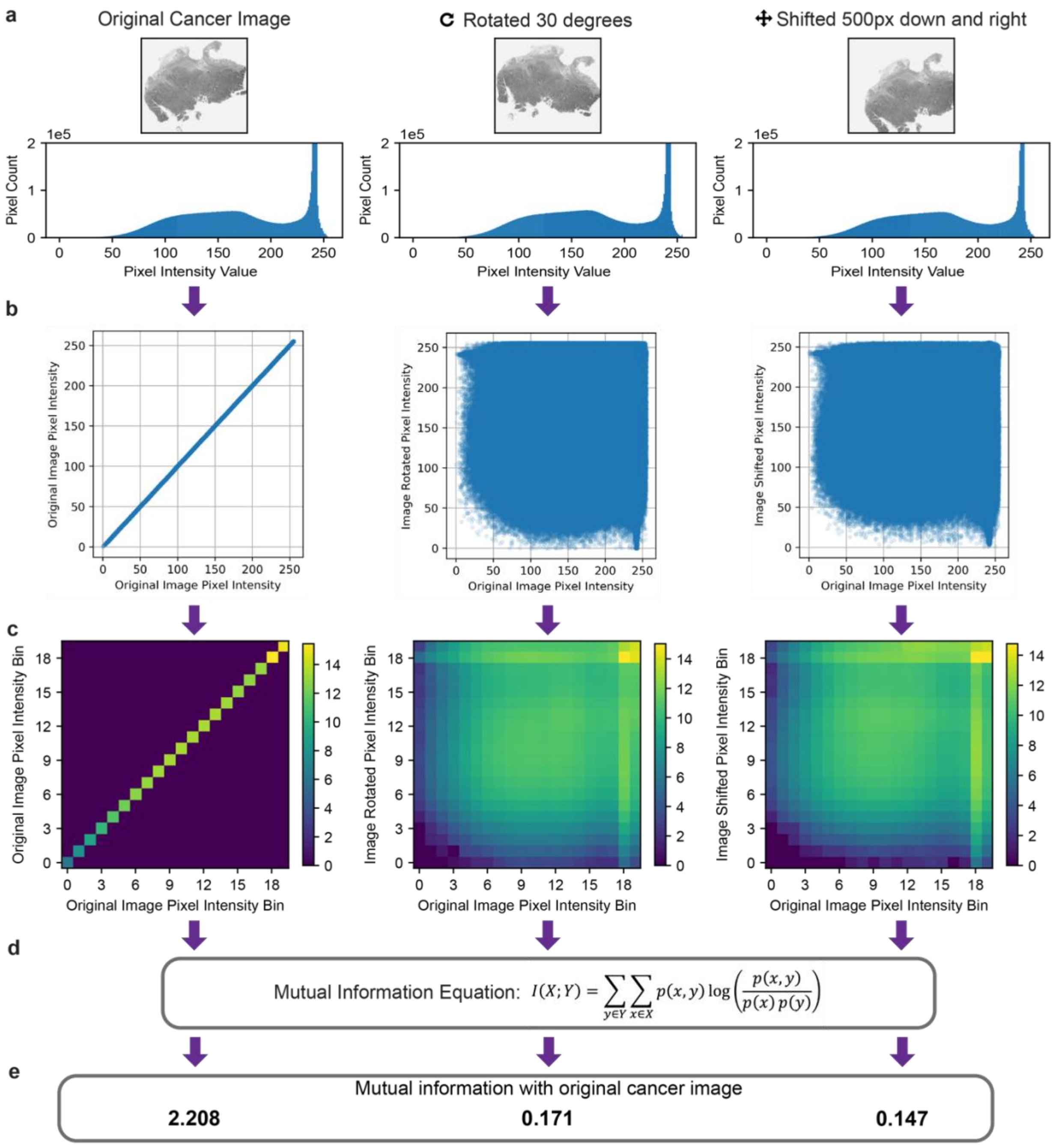

Supplemental Figure 3. **Mutual information measures image similarity**. Demonstration of mutual information calculation on images**. a,** Grayscale WSI images at 2x magnification with different transformations yet similar pixel intensity distributions. **b,** Visualization of joint probability distributions. **c,** Log scaled 2D heatmaps of joint probability distribution. **d,** Mutual information calculated using marginal and joint probability images. **e,** Mutual information between transformed images and original cancer image showing that mutual information is higher in aligned images.

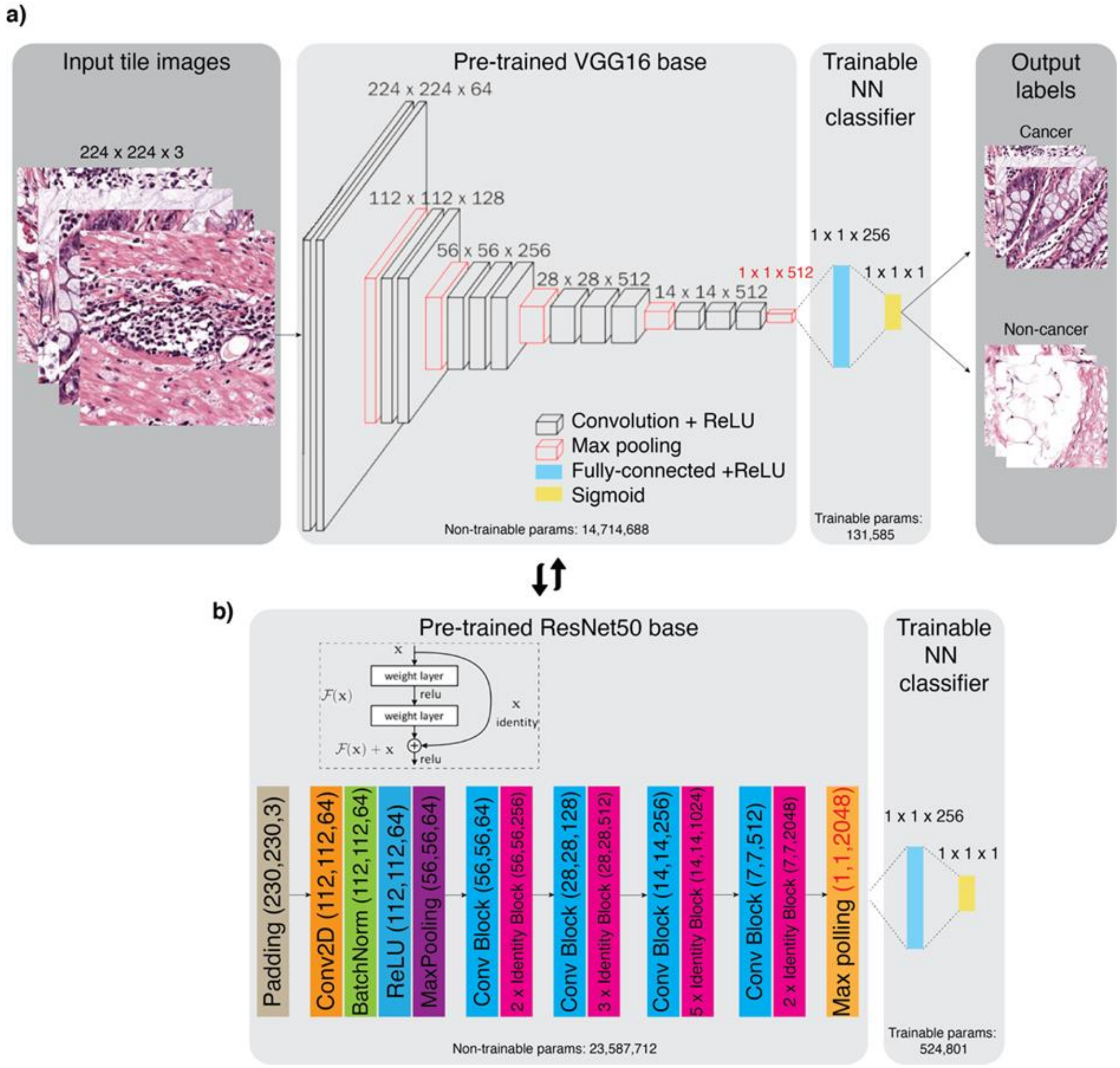

Supplemental Figure 4. **HEMnet model architecture**. a) HEMnet with VGG16 implemented. b) HEMnet with ResNet50 implemented (only hidden layers are shown).

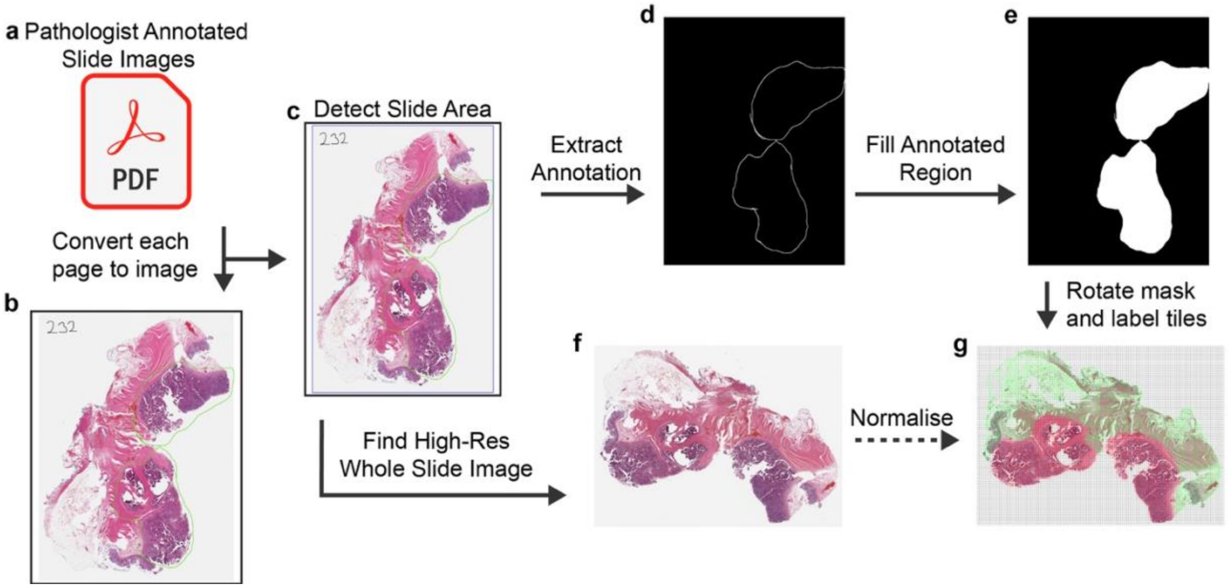

Supplemental Figure5. **Extraction of Pathologist Annotations. a,** PDF file containing pathologist annotations on low-resolution slide images. **b,** A single page with green pathologist annotations on a slide image. **c,** Detection of the bounds of slide image to remove white borders. **d,** Extraction of green cancer annotation line as a mask. **e,** Flood fill of annotation regions to form a pixel level cancer mask, upscaled to match size of high-resolution WSI. **f,** Identification of high-resolution WSI from low-resolution pdf image. **g,** Normalized and tiled WSI with each tile labelled as cancer (red) or non-cancer (green) based on pathologist cancer annotation mask.

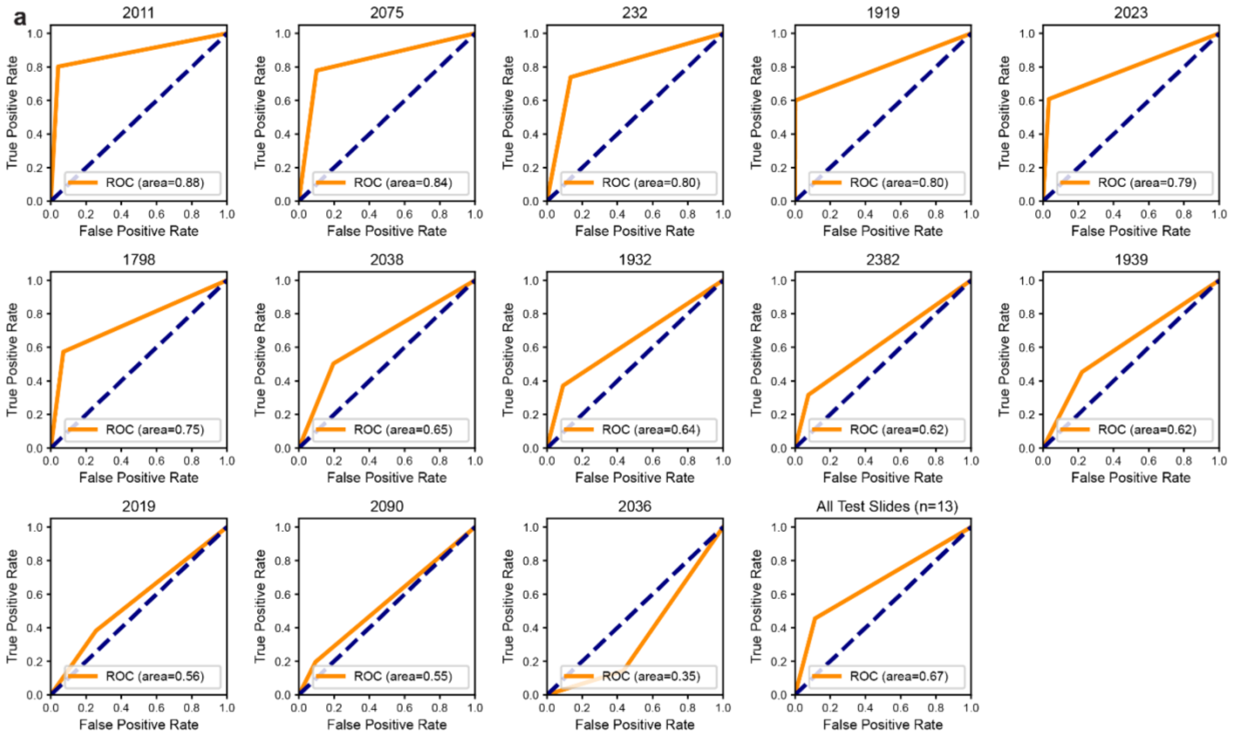

Supplemental Figure6. **p53 vs Pathologist annotations. a,** Receiver operating curves (ROC) for test slides tiles with labeled by p53 and Pathologist annotations (n=13) showing that p53 annotations often, but not always, agree with pathologist annotations. Each plot shows the ROC for tiles from a particular test slide, expect the last plot which shows the ROC for all test slides. Higher ROC area under the curve indicates more tiles labeled by p53 staining had the same cancer/non-cancer labels as tiles of the same location labeled by pathologist annotation.

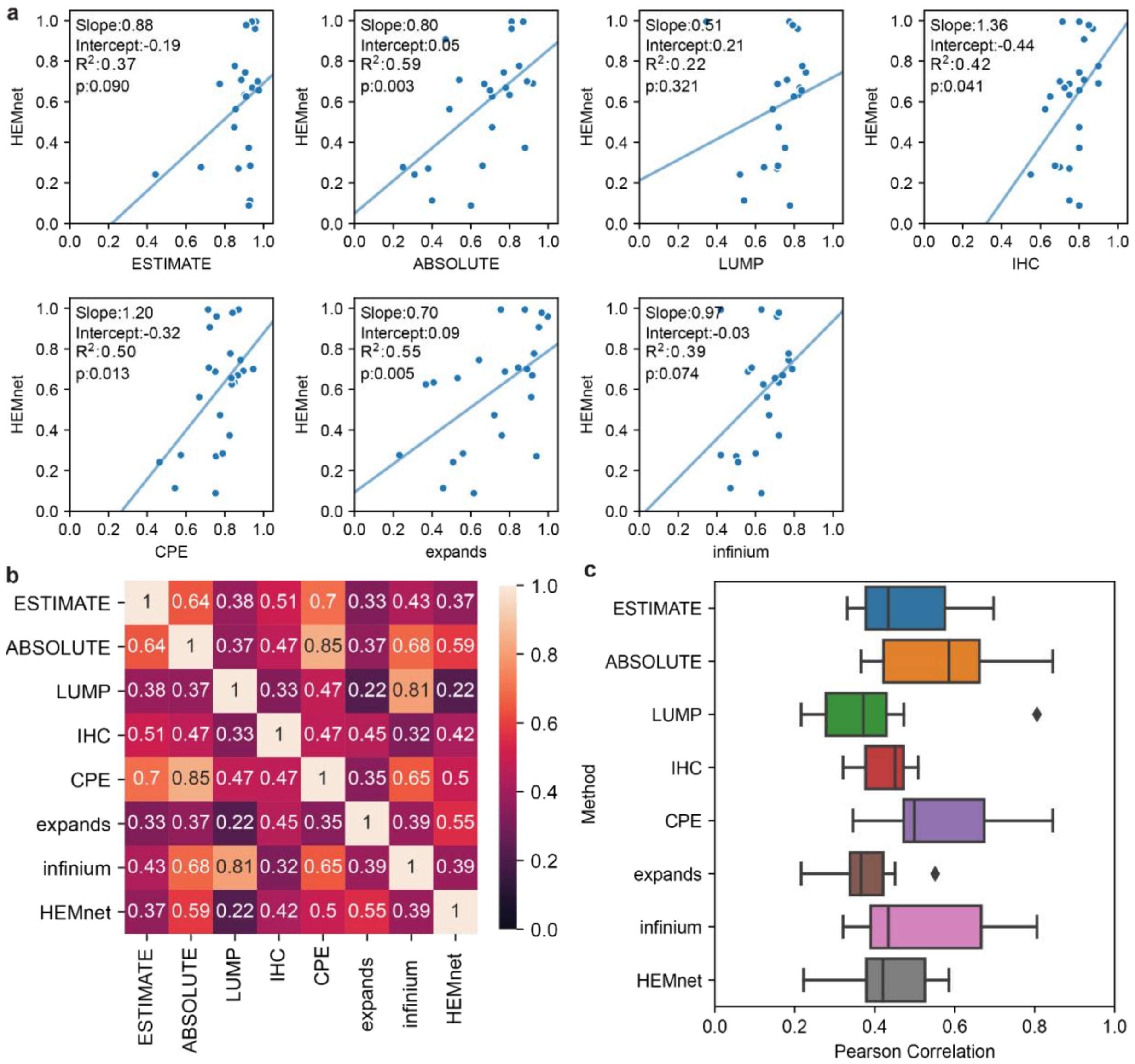

Supplemental Figure 7. **HEMnet vs sequencing estimates of tumour purity. a,** Individual plots of HEMnet cancer area proportion vs different sequencing estimates of tumour purity. **b,** Pearson correlations between different estimates of tumour purity. **c,** Boxplot of Pearson correlations between one method against all other methods. HEMnet performs similarly to other methods.

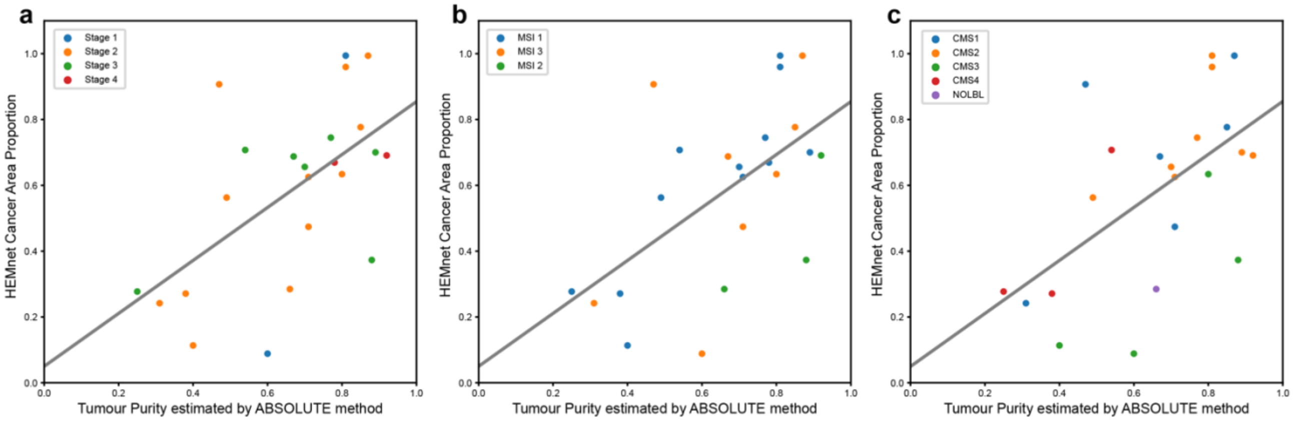

Supplemental Fig. S8. **Assessing prediction performance based on different cancer stages.**

MSI: microsatellite instability status. CMS: consensus molecular subgroup.

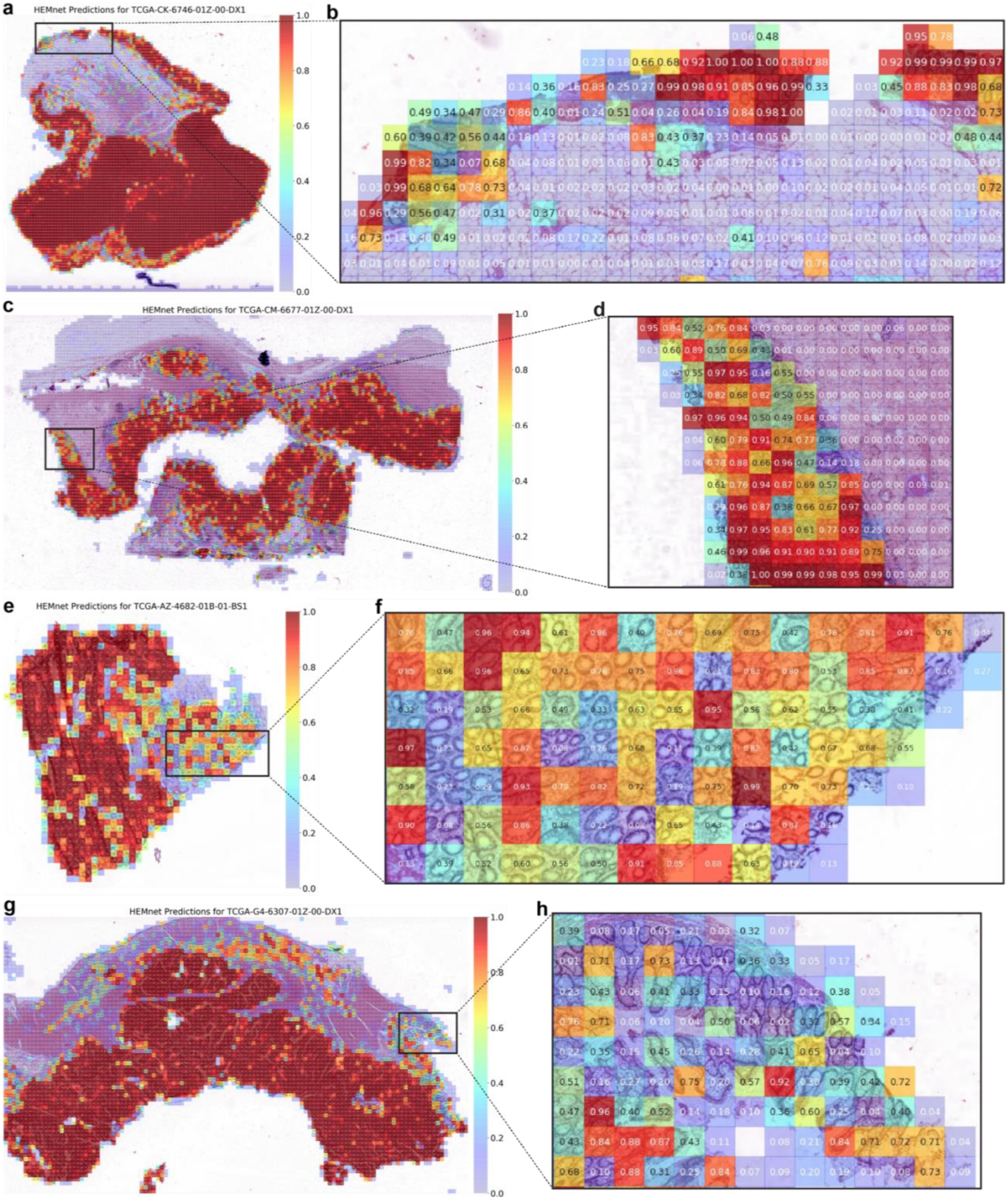

Supplemental Fig. S9. **HEMnet prediction outputs for normal colon epithelium in TCGA samples. a, c, e, g** Prediction output heatmaps for TCGA images used for external validation (0 suggests normal and 1 suggests cancer). **b, d, f, h** Zoomed in prediction output heatmaps of normal colon epithelium showing uncertain regions (predictions around 0.5) and annotated by individual tile prediction outputs which could help pathologists review slides.

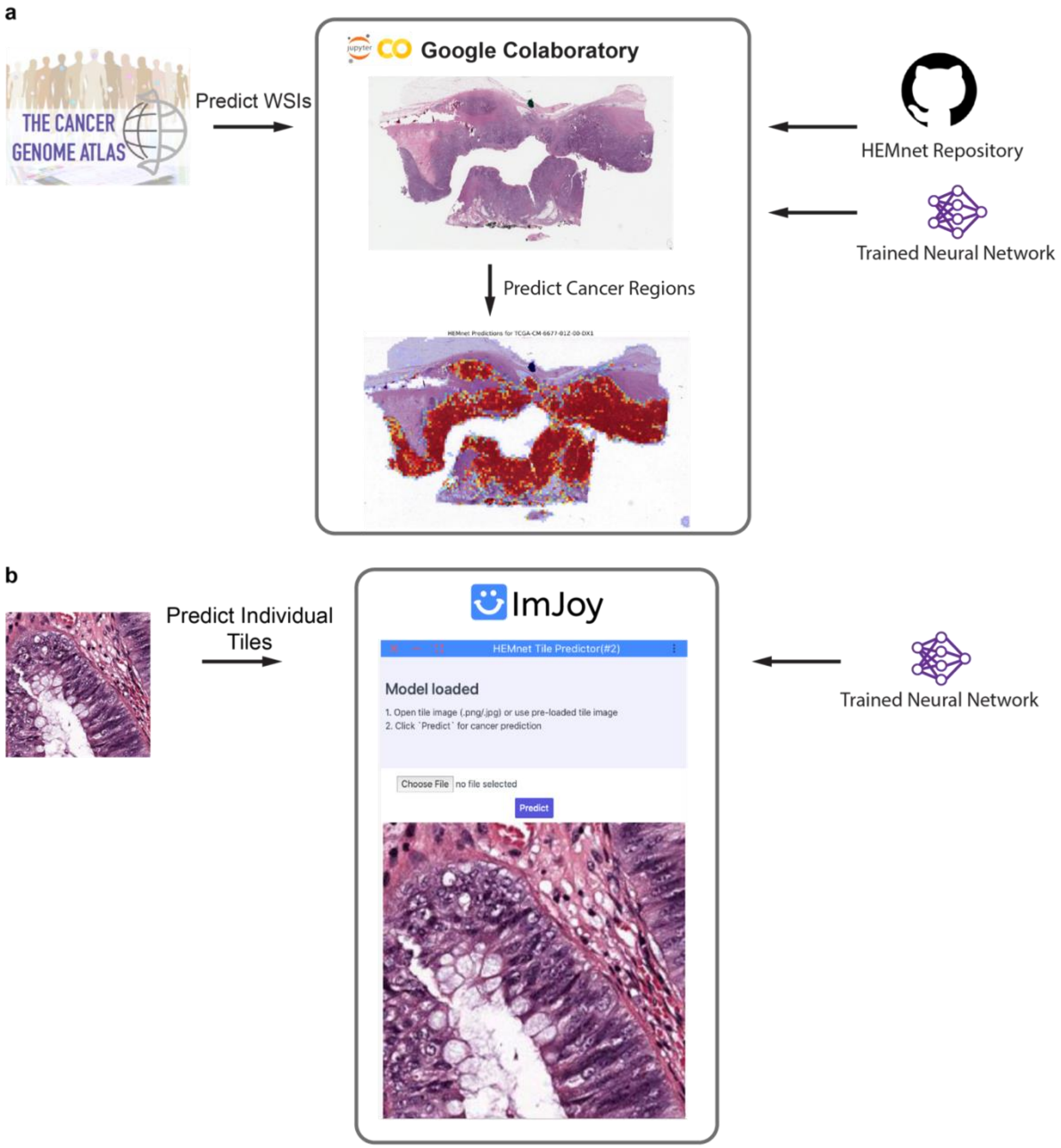

Supplemental Fig. S10. **HEMnet Demos in the cloud. a,** Google colaboratory notebook for using HEMnet and our trained colorectal cancer model to predict on slides from TCGA. Outputs predicted cancer regions and estimated cancer area proportion. **b,** ImJoy plugin for predicting on individual tiles using our trained colorectal cancer model.

Supplemental Tables:

Supplementary Table 1. **Comparison of model architectures on validation performance**

Model	Accuracy*	AUC*	F-score*	Runtime^
VGG16	0.81	0.73	0.76	600 min
VGG19	0.8	0.73	0.73	754 min
ResNet50	0.77	0.5	0.67	725 min
Inception v3	0.76	0.61	0.76	600 min
Xception	0.77	0.51	0.68	990 min

*Scores calculated from performance on 5 validation slides

^Runtime calculated from training time (on 10 WSIs) + validation inference time for 100 epochs with early stopping using 2 GPUs (NVIDIA Tesla V100-PCIE-16GB)

Supplementary Table 2. Validation results using TCGA Colon Adenocarcinoma (COAD) dataset.

Sample IDs	WSI Area (mm^2)	Tissue Area (mm^2)	Total Tiles	Cancer Tiles	HEMnet* Cancer Tile Proportion	HEMnet Cancer Area Proportion	ESTIMATE*	ABSOLUTE*	LUMP*	IHC*	CPE*	EXPANDS*	infinium*	TP53 Mutation Type	Clinical Stage	MSI Status	CMS-RFclassifier
A6-5656	261.26	18.58	529	470	0.888	0.994	0.9606	0.81	0.774	0.714	0.714	0.879	0.63	Missense_ Mutation	1	1	CMS2
A6-6650	448.67	76.96	1901	1633	0.859	0.960	0.9562	0.81	0.818	0.871	0.757	0.998	0.71	Missense_ Mutation	2	1	CMS2
AA-3715	318.48	74.11	7886	6533	0.828	0.907	NA	0.47	NA	0.825	0.722	0.951	NA	None	2	3	CMS1
AA-3973	127.58	44.20	4919	3363	0.684	0.691	NA	0.92	NA	0.900	0.896	0.898	NA	None	4	2	CMS2
AD-6889	220.43	121.54	3032	2764	0.912	0.994	0.9399	0.87	0.346	0.800	0.871	0.755	0.42	3'UTR	2	3	CMS1
AZ-4615	560.41	235.96	6421	3640	0.567	0.688	0.7739	0.67	0.713	0.750	0.751	0.776	0.56	None	3	3	CMS1
AZ-4682	539.76	174.56	5569	2602	0.467	0.669	0.9415	0.78	0.827	0.725	0.867	0.918	0.74	None	4	1	
AZ-5403	852.98	638.19	13865	3567	0.257	0.271	0.87	0.38	0.711	0.750	0.754	0.939	0.5	Missense_ Mutation	2	1	CMS4
CA-6715	395.61	232.72	5840	3560	0.610	0.700	0.9696	0.89	0.762	0.700	0.947	0.890	0.79	Missense_ Mutation	3	1	CMS2
CK-5913	488.02	291.19	7160	3044	0.425	0.474	0.8486	0.71	0.718	0.800	0.776	0.721	0.67	None	2	3	CMS1
CK-5914	504.17	221.81	5200	3705	0.713	0.745	0.9052	0.77	0.859	0.800	0.882	0.643	0.77	Missense_ Mutation	3	1	CMS2
CK-6746	436.20	232.33	5409	3753	0.694	0.777	0.852	0.85	0.842	0.900	0.829	0.926	0.77	None	2	3	CMS1
CK-6747	580.80	265.62	6853	3680	0.537	0.634	0.9021	0.8	0.821	0.750	0.851	0.408	0.72	None	2	3	CMS3
CM-5344	546.99	312.38	7301	4771	0.653	0.707	0.8858	0.54	0.763	0.825	0.718	0.845	0.58	None	3	1	CMS4
CM-6167	472.38	261.19	6217	1567	0.252	0.277	0.6777	0.25	0.644	0.700	0.573	0.231	0.42	Missense_ Mutation	3	1	CMS4
CM-6171	863.72	294.10	7046	6462	0.917	0.978	0.9107	NA	0.792	0.850	0.840	0.966	0.72	None	1	3	CMS1
CM-6677	678.28	292.76	7136	3443	0.482	0.563	0.8565	0.49	0.688	0.625	0.668	0.912	0.66	Missense_ Mutation	2	1	CMS2
D5-5537	953.17	240.05	7030	3210	0.457	0.625	0.9103	0.71	0.798	0.650	0.836	0.367	0.64	None	2	1	CMS2
D5-6928	84.49	43.36	1003	245	0.244	0.242	0.4425	0.31	0.520	0.550	0.464	0.508	0.51	None	2	3	CMS1
DM-A0XD	176.37	45.13	1453	306	0.211	0.285	0.9314	0.66	0.715	0.675	0.789	0.560	0.6	Missense_ Mutation	2	2	NOLBL
DM-A280	860.89	398.12	11938	1229	0.103	0.113	0.9301	0.4	0.540	0.750	0.542	0.457	0.47	None	2	1	CMS3
F4-6856	424.60	174.41	4971	385	0.077	0.088	0.9244	0.6	0.777	0.800	0.752	0.616	0.63	Nonsense_ Mutation	1	3	CMS3
G4-6307	410.88	215.09	5228	3369	0.644	0.656	0.9759	0.7	0.837	0.800	0.835	0.532	0.7	Missense_ Mutation	3	1	CMS2
G4-6322	539.83	283.59	8410	2300	0.273	0.373	0.9237	0.88	0.752	0.800	0.825	0.760	0.72	None	3	2	CMS3

*HEMnet tumour purity prediction results are compared to seven genomics based methods
