# Supplementary material for: A deep learning model for molecular label transfer that enables cancer cell identification from histopathology images
Source: NPJ Precis Oncol. 2022 Mar 2;6:14. doi: 10.1038/s41698-022-00252-0 (PMC8891271; doi:10.1038/s41698-022-00252-0)
Supplement: Supplementary file 2 — REPORTING SUMMARY [file 41698_2022_252_MOESM2_ESM.pdf]

nature portfolio

Corresponding author(s): Quan Hoang Nguyen

Last updated by author(s): 2021/07/14

Reporting Summary

Nature Portfolio wishes to improve the reproducibility of the work that we publish. This form provides structure for consistency and transparency in reporting. For further information on Nature Portfolio policies, see our Editorial Policies and the Editorial Policy Checklist.

Statistics

For all statistical analyses, confirm that the following items are present in the figure legend, table legend, main text, or Methods section.

n/a	Confirmed	
☐	☒	The exact sample size (n) for each experimental group/condition, given as a discrete number and unit of measurement
☐	☒	A statement on whether measurements were taken from distinct samples or whether the same sample was measured repeatedly
☐	☒	The statistical test(s) used AND whether they are one- or two-sided<br>*Only common tests should be described solely by name; describe more complex techniques in the Methods section.*
☐	☒	A description of all covariates tested
☐	☒	A description of any assumptions or corrections, such as tests of normality and adjustment for multiple comparisons
☐	☒	A full description of the statistical parameters including central tendency (e.g. means) or other basic estimates (e.g. regression coefficient) AND variation (e.g. standard deviation) or associated estimates of uncertainty (e.g. confidence intervals)
☐	☒	For null hypothesis testing, the test statistic (e.g. F, t, r) with confidence intervals, effect sizes, degrees of freedom and P value noted<br>*Give P values as exact values whenever suitable.*
☐	☒	For Bayesian analysis, information on the choice of priors and Markov chain Monte Carlo settings
☐	☒	For hierarchical and complex designs, identification of the appropriate level for tests and full reporting of outcomes
☐	☒	Estimates of effect sizes (e.g. Cohen's d, Pearson's r), indicating how they were calculated

Our web collection on statistics for biologists contains articles on many of the points above.

Software and code

Policy information about availability of computer code

Data collection	All digital slide images were generated in Aperio SVS format by Translational Pathology Core Laboratory at University of California, Los Angeles. This study was conducted in compliance with the Helsinki Declaration. Each tissue section was scanned at 20x magnification to generate a total of 35 of p53 and H&E pairs of high resolution WSIs.
Data analysis	The source code, tutorials and interactive analysis tools are available at https://github.com/BiomedicalMachineLearning/HEMnet. We also provide cloud-based implementation of the HEMnet (Figure S8), available as Google Colab notebook and an ImJoy application (links to these apps are on HEMnet github page). HEMnet is also available as an open-source PyPI python package (https://pypi.org/project/hemnet).

For manuscripts utilizing custom algorithms or software that are central to the research but not yet described in published literature, software must be made available to editors and reviewers. We strongly encourage code deposition in a community repository (e.g. GitHub). See the Nature Portfolio guidelines for submitting code & software for further information.

Data

Policy information about availability of data

All manuscripts must include a data availability statement. This statement should provide the following information, where applicable:

- Accession codes, unique identifiers, or web links for publicly available datasets
- A description of any restrictions on data availability
- For clinical datasets or third party data, please ensure that the statement adheres to our policy

The datasets used and/or analysed during the current study are available from the https://dna-discovery.stanford.edu/research/web-resources/HEMnet.

Field-specific reporting

Please select the one below that is the best fit for your research. If you are not sure, read the appropriate sections before making your selection.

☒ Life sciences ☐ Behavioural & social sciences ☐ Ecological, evolutionary & environmental sciences

For a reference copy of the document with all sections, see nature.com/documents/nr-reporting-summary-flat.pdf

Life sciences study design

All studies must disclose on these points even when the disclosure is negative.

Sample size	We obtained 32 high-resolution H&E images and corresponding p53 IHC images from 27 cancer samples and 5 non-cancer samples. First, the pixel-level labels allow us to divide one image into hundreds to thousands of smaller, high-resolution, molecular labelled tiles, thereby increasing sample sizes for model training and testing. We were confident that cells were correctly labelled, with 8,782 non-cancer tiles and 21,939 cancer tiles.
Data exclusions	Similar to other IHC markers, p53 staining has its limitations as within one image or between images, the marker is not always indicative of cancer, vice versa. For example, overexpression and positive staining for p53 may occur in normal cells responding to DNA damage. In addition p53 may be absent in cancer cells with TP53 gene deletions. To overcome these limitations, when training our model, we only considered p53 positive cells as cancer if they come from a cancer slide and only p53 negative cells from slides where the cells have a normal morphology. We removed 23,275 tiles that had some levels of uncertainty in
Replication	As an independent validation using an external dataset, we applied our method HEMnet to colon adenocarcinoma samples from TCGA colon cancer samples.
Randomization	Random selection was applied using k-fold cross validation while training the model.
Blinding	The investigators were blind against the comparison results from TCGA genomics analysis

Reporting for specific materials, systems and methods

We require information from authors about some types of materials, experimental systems and methods used in many studies. Here, indicate whether each material, system or method listed is relevant to your study. If you are not sure if a list item applies to your research, read the appropriate section before selecting a response.

Materials & experimental systems

n/a	Involved in the study	
☐	☒	Antibodies
☒	☐	Eukaryotic cell lines
☒	☐	Palaeontology and archaeology
☒	☐	Animals and other organisms
☐	☒	Human research participants
☒	☐	Clinical data
☒	☐	Dual use research of concern

Methods

n/a	Involved in the study	
☒	☐	ChIP-seq
☒	☐	Flow cytometry
☒	☐	MRI-based neuroimaging

Antibodies

Antibodies used	IHC staining against p53 using DO-7 monoclonal antibody (Roche) by Anatomic Pathology & Clinical Laboratories at Stanford Medicine.
Validation	Validation of positive antibody staining was based on comparisons with manual pathologist's annotation.

Human research participants

Policy information about studies involving human research participants

Population characteristics	We collected cancer tissue samples from 30 patients at Stanford Hospital. Tissues were obtained from the Stanford Cancer Institute Tissue Bank. In addition, we obtained matched normal, non-cancer tissue from five patients.
Recruitment	All patients were enrolled according to a study protocol approved by the Stanford University School of Medicine Institutional Review Board (IRB-11886). All participants provided written informed consent to take part in the study.
Ethics oversight	Study protocol was approved by the Stanford University School of Medicine Institutional Review Board (IRB-11886)

Note that full information on the approval of the study protocol must also be provided in the manuscript.
